# Integrated mRNA and miRNA expression profiling in blood reveals candidate biomarkers associated with endurance exercise in the horse

**DOI:** 10.1038/srep22932

**Published:** 2016-03-10

**Authors:** Núria Mach, Sandra Plancade, Alicja Pacholewska, Jérôme Lecardonnel, Julie Rivière, Marco Moroldo, Anne Vaiman, Caroline Morgenthaler, Marine Beinat, Alizée Nevot, Céline Robert, Eric Barrey

**Affiliations:** 1 Animal Genetics and Integrative Biology unit (GABI), INRA, AgroParis Tech, Université Paris-Saclay, 78350, Jouy-en-Josas, France; 2 INRA, MaIAGE, Jouy-en-Josas, France; 3 Swiss Institute of Equine Medicine, Institute of Genetics, University of Bern and Agroscope, Bern, Switzerland; 4 Université Paris-Est, Ecole Vétérinaire d’Alfort, Maisons-Alfort, France; 5 Integrative Biology and Exercice Adaptation unit (UBIAE), EA7362, Université d’Evry Val d’Essonne, Evry, France

## Abstract

The adaptive response to extreme endurance exercise might involve transcriptional and translational regulation by microRNAs (miRNAs). Therefore, the objective of the present study was to perform an integrated analysis of the blood transcriptome and miRNome (using microarrays) in the horse before and after a 160 km endurance competition. A total of 2,453 differentially expressed genes and 167 differentially expressed microRNAs were identified when comparing pre- and post-ride samples. We used a hypergeometric test and its generalization to gain a better understanding of the biological functions regulated by the differentially expressed microRNA. In particular, 44 differentially expressed microRNAs putatively regulated a total of 351 depleted differentially expressed genes involved variously in glucose metabolism, fatty acid oxidation, mitochondrion biogenesis, and immune response pathways. In an independent validation set of animals, graphical Gaussian models confirmed that miR-21-5p, miR-181b-5p and miR-505-5p are candidate regulatory molecules for the adaptation to endurance exercise in the horse. To the best of our knowledge, the present study is the first to provide a comprehensive, integrated overview of the microRNA-mRNA co-regulation networks that may have a key role in controlling post-transcriptomic regulation during endurance exercise.

The physiological and biochemical demands of endurance exercise elicit both muscle-based and systemic responses. The main adaptations to endurance exercise include improvement of mechanical, metabolic, neuromuscular and contractile functions in muscle^1^, correction of electrolyte imbalance^2^, a decrease in glycogen storage^3^ and an increase in mitochondrial biogenesis in muscle tissue^4^, and the modulation of oxidative stress^5^, intestinal permeability, muscle damage, systemic inflammation and immune responses^5^. Consequently, adaptations to endurance exercise are influenced by the transcriptional and translational regulation of genes that encode the proteins controlling these processes^5^. Over the past decade, microRNAs (miRNAs) have emerged as novel elements in the rapid, reversible regulation of transcription and translation^6^. MiRNAs are small non-coding RNAs molecules (~19–24 bp in length) that are synthesized from short hairpin precursors and that reportedly degrade or inhibit the translation of their target genes by binding to the 3′ untranslated region (UTR) of coding mRNAs^7^. In fact, miRNAs molecules may regulate up to one-third of the mammalian transcriptome^8^ and appear to be stable outside the cell (e.g. when incorporated into exosomes^9^, microvesicles^10^, lipoproteins^11^ or Argonaute2 protein complexes^12^).

It has been shown that some miRNAs can modulate mitochondrial biogenesis and glucose and fatty acid metabolism in skeletal and cardiac muscle after endurance exercise in humans^1^,^13^. Furthermore, circulating miRNAs induced by exhaustive exercise in humans may have an important role in the regulation of angiogenesis, inflammation, skeletal and cardiac muscle contractility and damage, and adaptation to hypoxia/ischaemia^13^,^14^,^15^,^16^,^17^,^18^,^19^, whereas miRNA expression in peripheral blood mononuclear cells (PBMCs) may regulate inflammatory processes^20^,^21^. Whole blood-derived miRNAs induced in trained athletes after a 30-minute treadmill test may regulate immune function, apoptosis, membrane traffic of proteins and transcription regulation^22^. Whole blood-derived miRNAs that control the stress response to endurance exercise have never previously been studied. However, we hypothesized that whole blood (which contains cells that circulate throughout the body) might be able to provide a rapidly measureable, effective marker of an athlete’s immune function and might also be sensitive enough to detect exercise-induced stress and metabolic disorders.

Given the miRNAs’ fundamental role in transcription and transcriptional regulation during physiological adaptation to endurance exercise, we hypothesized that the identification of whole blood miRNA-mRNA relationships specifically regulated by endurance exercise in horses could (i) reveal unique biomarkers of the stress response to endurance exercise and (ii) provide significant insights into the molecular control of this response. We therefore performed an integrated analysis of the blood transcriptome and miRNome in the horse before and after a long (160 km) endurance competition.

## Results

The morphological and physiological parameters of the 61 equine athletes’ included in the study are summarized in Supplementary Table S1, whereas the biochemical parameters average obtained from blood samples collected after the ride are depicted in the Supplementary Table S2. All horses had an above-normal total bilirubin, creatine kinase, aspartate transaminase and serum amyloid A concentrations, reflecting haemolysis and muscular membrane permeability or inflammation.

**Figure 1:**
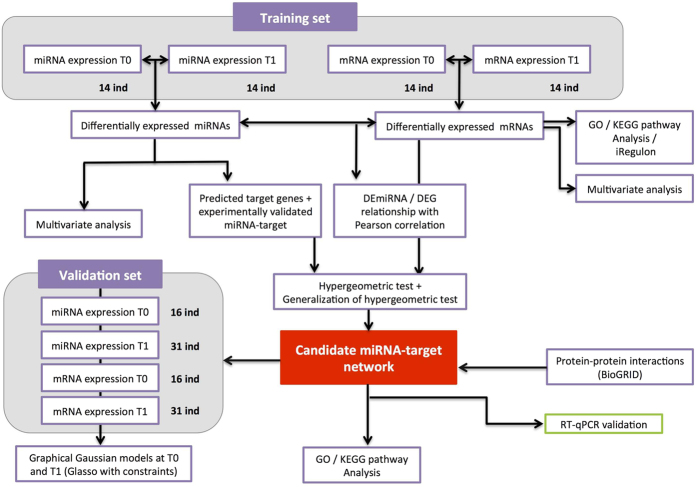
Overview of the data analysis. Step 1: a linear model analysis of DEmiRNAs and DEGs. Step 2: determination of the corresponding GO terms, KEGG pathways and TFs regulating the DEGs. Step 3: selection of experimentally validated target genes. Step 4: generation of the inverse correlation matrix for DEGs and miRNAs (in the hypergeometric test only). Step 5: the enrichment test (using the hypergeometric test or its generalization) used to select candidate miRNAs. Step 6: the functional network analysis; Step 7: validation of the functional network, using a validation set and GGMs; Step 8: Technical validation of the functional network, using RT-qPCR.

On the basis of equine transcriptome and miRNome profiles before and after a 160 km ride, we used a multi-step approach to identify and characterize a dynamically co-regulated miRNA-mRNA network related to endurance exercise in the horse (Fig. 1).

### Differentially expressed genes (DEGs) and miRNAs

We used custom equine mRNA and miRNA microarrays to study the effect of endurance exercise on the blood transcriptome and miRNome, respectively, using an experimental set of 14 animals. We focused on the identification of genes whose expression was significantly altered at T1 (post-ride) relative to T0 (baseline, pre-ride), with an adjusted *p*-value < 0.05 after Bonferroni correction for multiple testing (Supplementary Table S3). Application of this threshold led to the identification of 2,453 DEGs, of which 1,165 were over-expressed and 1,288 were under-expressed at T1 (relative to T0).

**Figure 2:**
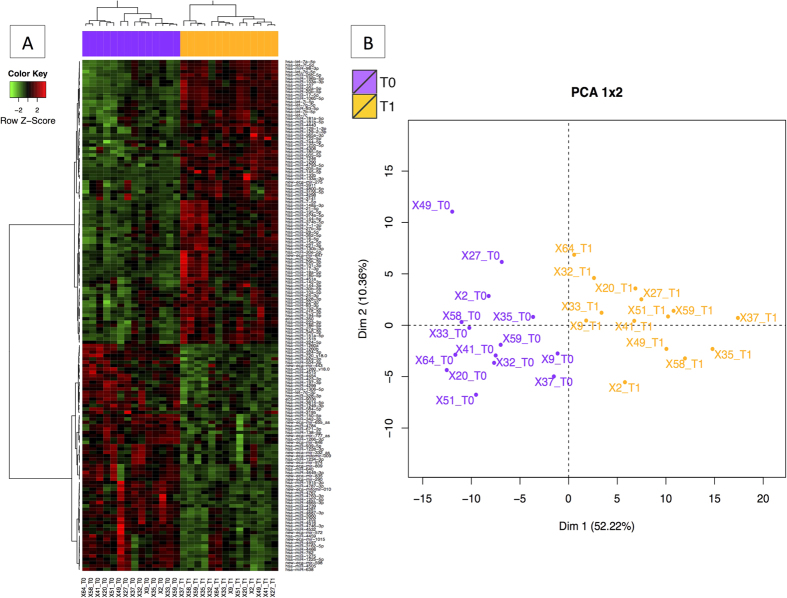
The differential miRNA expression profile in blood. In all cases, individual horses are represented as violet dots (for *T0*) and orange dots (for *T1*). (**A**) A heat-map representation of the 167 DEmiRNAs when comparing T0 and T1 (FDR < 0.05). (**B**) PCA of DEmiRNAs in blood when comparing T1 with T0 (FDR < 0.05). The first axis accounted for 52.22% of the variation, and the two axes accounted for 62.58% of the total inertia. The two groups were found to differ significantly in a Monte Carlo test with 999 replicates (*p* < 0.001).

A total of 362 miRNAs were found in the whole blood sample (Supplementary Table S4). We identified differentially expressed 167 miRNAs (DEmiRNAs) when comparing pre- and post-ride samples (Supplementary Table S5). Along with orthologous human-equine DEmiRNAs, we detected 12 equine-specific DEmiRNAs and 19 putative novel miRNAs. Two of the putative novel miRNAs (mitomiR-009 and mitomiR-010) were probably encoded by the mitochondrial genome and thus are referred to here as mitomiRs (Supplementary Fig. S1). The effect of exercise on the miRNome was also evidenced by a cluster analysis and a principle component analysis (PCA) (Fig. 2). The first principal component accounted for 52.22% of the total variance, and the first two components accounted for 62.58% of the total variance.

**Figure 3:**
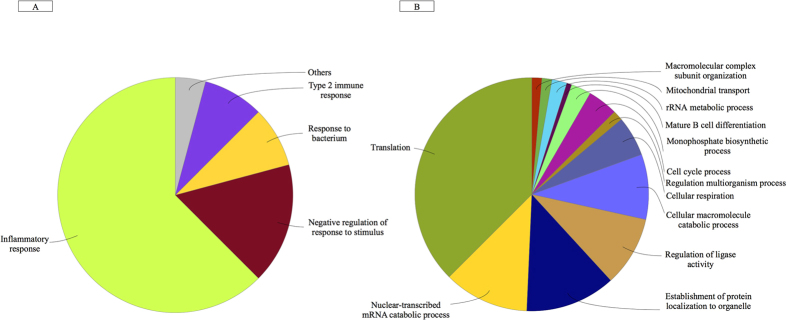
A functional map of DEGs, showing the top categories of GO biological processes associated with significantly over-expressed (**A**) and under-expressed (**B**) genes following endurance exercise. The chart fragments represent the number of genes associated with the various terms.

**Figure 4:**
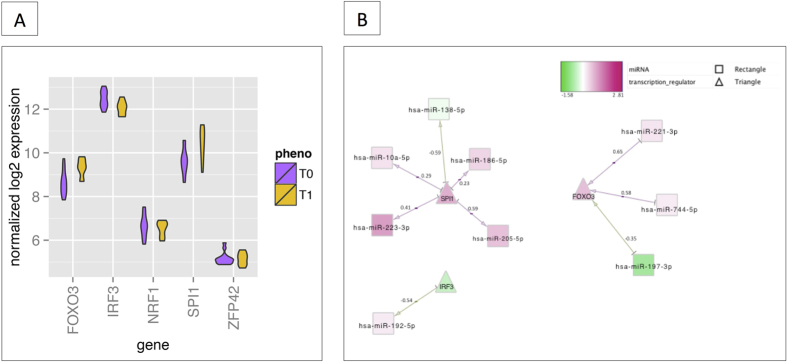
Relationship between TFs, miRNAs and DEGs. (**A**) The boxplot graph represents the expression levels (log2) of the main candidate TFs (discovered using iRegulon) within the set of DEGs. *ZFP42* was the top regulator, followed by *SPI1, FOXO3, IRF3* and *NRF1*. (**B**) A schematic view of the common correlations between TFs and miRNAs. The correlation score for expression of the two interactants is indicated under each edge. The network is displayed graphically as nodes (genes, TFs and miRNAs) and edges (biological relationships). The edge colour intensity indicates the expression level of the association: red = over-expression at T1 and green = under-expression at T1. The node shape indicates whether the node is a TF (triangles) or a miRNA (squares).

To gain a better understanding of the functional implications of these DEGs, we performed gene set enrichment analysis in order to identify over-represented “biological process” gene ontology (GO) terms and we characterized the transcription factors (TFs) that regulate DEGs. The 1,165 DEGs over-expressed after exercise were strongly associated with the inflammatory response, intestinal permeability and regulation of the response to stress and bacterium (Fig. 3A). In contrast, the 1,288 under-expressed DEGs were related to macromolecule catabolism, cellular respiration, mitochondrial transport, and transcriptional and translational activity (Fig. 3B). The full list of significantly enriched pathways (false discovery rate (FDR) < 0.001) is given in Supplementary Table S6. The main putative regulatory TF in the network of DEGs was *ZFP42*, followed by the cooperatively transcriptional cofactors *SPI1, FOXO3, IRF3* and *NRF1* (Fig. 4). TFs act in regulatory networks and can drive or repress the expression of the miRNAs in a feed-forward and feedback manner^23^. Accordingly, we found that these TFs might control the spatiotemporal expression patterns of some of the DEmiRNAs (Fig. 4B).

The Supplementary Information section contains further details of (i) the characterization of transcription factors that regulate differentially expressed genes and (ii) the validation of mRNA and miRNA expression experiments using RT-qPCR.

### Target DEGs were inversely correlated with functionally enriched miRNAs

We used two complementary statistical approaches (detailed in the Methods section) to identify the target-enriched miRNAs and assess their role in regulating the exercise response. Firstly, for each of the 167 DEmiRNAs, we used the multiMiR package in R to build a comprehensive list of all the putative validated target genes. We found that only 91 DEmiRNAs (70 of which were up-regulated and 21 of which were down-regulated in post-ride samples, compared with pre-ride samples) presented an experimentally annotated targetome. In total, these DEmiRNAs regulated 7,150 putative target genes-most of which were down-regulated after the ride (Supplementary Table S7). For each of the 91 DEmiRNAs presenting at least one experimentally validated target, we looked at whether the DEGs that were significantly and negatively correlated with DEmiRNAs were in fact miRNA targets. The hypergeometric test revealed two DEmiRNAs (miR-138-5p and miR-26b-5p) with significantly more predicted targets among the negatively correlated DEGs than among other genes in the transcriptome (FDR < 0.10; Supplementary Table S8). Next, to address the hypergeometric test’s lack of power (given that there were few anticorrelated target genes for a given DEmiRNA), we applied a generalization of the hypergeometric test to each of the 91 DEmiRNAs. The objective was to establish whether the experimentally validated target genes displayed smaller Limma *p*-values for differential expression (when comparing pre- and post-ride samples) than other genes in the transcriptome (according to a Wilcoxon sum rank test). This generalization of the hypergeometric test also encompassed targets affected by translation inhibition, i.e. targets for which changes in miRNA levels do not reduce mRNA levels (r > 0.5) but do reduce the protein levels^24^. We observed 45 enriched DEmiRNAs (FDR < 0.1; Supplementary Table S8) and then selected those for which the target genes were DEGs and were inversely correlated (r^2^ < −0.5). This calculation removed the hsa-miR-374a-5p and hsa-miR-374b-5p from the list, since their targets were DEGs but were not greatly anticorrelated (r > −0.5).

**Figure 5:**
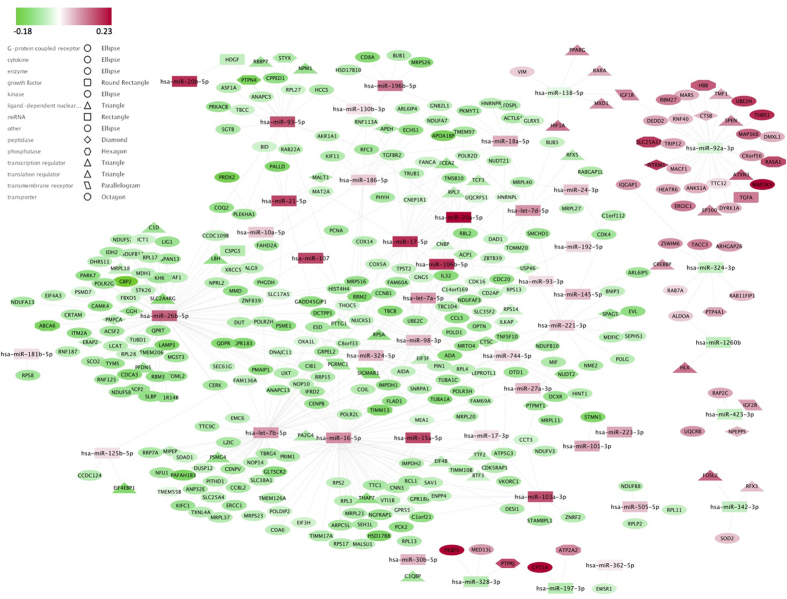
Regulatory network linking the 44 enriched miRNA and their respective inversely correlated target DEGs. We identified a total of 44 enriched DEmiRNAs for which the miRNA targets defined by multimiR were significantly enriched (according to a hypergeometric test or its generalization). The 44 DEmiRNAs were inversely correlated with a total of 351 unique target DEGs. The network is displayed graphically as nodes (genes and miRNAs) and edges (biological relationships). The edge colour intensity indicates the expression level (log2) of the association: red = over-expression at T1 and green = under-expression at T1. The node shape indicates whether the node is a transcription regulator (triangles), growth factor (round rectangles), peptidase (diamonds), phosphatase (hexagons), transmembrane receptor (parallelograms), transporter (octagons), a miRNA (squares) or other type of genes (ellipses).

Ultimately, we considered a total of 44 enriched miRNAs (Supplementary Fig. S2; Supplementary Table S9) inversely correlated with the expression of 351 target DEGs during exercise (Supplementary Table S10; Fig. 5). We next examined the protein-protein interaction (PPI) sub-network associated with the 44 enriched miRNAs and their inversely correlated target DEGs (see Supplementary Information Section).

**Figure 6:**
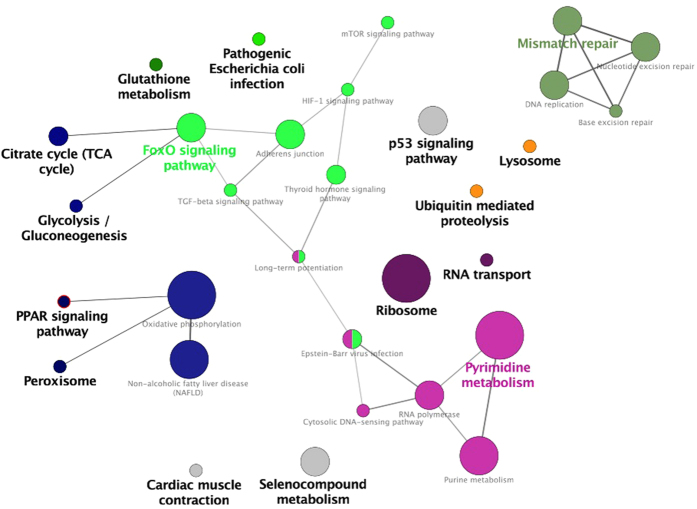
A functional map of the 351 inversely correlated target genes regulated by the 44 enriched miRNAs. Within the network, the GO biological terms were identified as nodes and then linked according to their kappa value (>0.4) and FDR (<0.001). The size of the node corresponds to the statistical significance of the enrichment term. Functionally related groups partially overlap. Similar GO terms are labelled in the same colour. Non-grouped terms are shown in grey. The colour gradient shows the proportion of genes in each cluster associated with the term.

To identify the most relevant cellular activities controlled by these 351 anticorrelated target DEGs, we analysed overrepresented GO biological process terms (using clueGO). The most significant GO terms were related to glucose metabolism, fatty acid oxidation, mitochondrial biogenesis trough the peroxisome proliferator-activated receptor (PPARγ) signalling pathway, oxidation stress, proteolysis and immune response pathways (Fig. 6). To provide further insight into proteolysis and immune response pathways, we also analysed the correlations between changes in the enriched DEmiRNA levels and biochemical blood parameters (see Supplementary Information Section).

**Figure 7:**
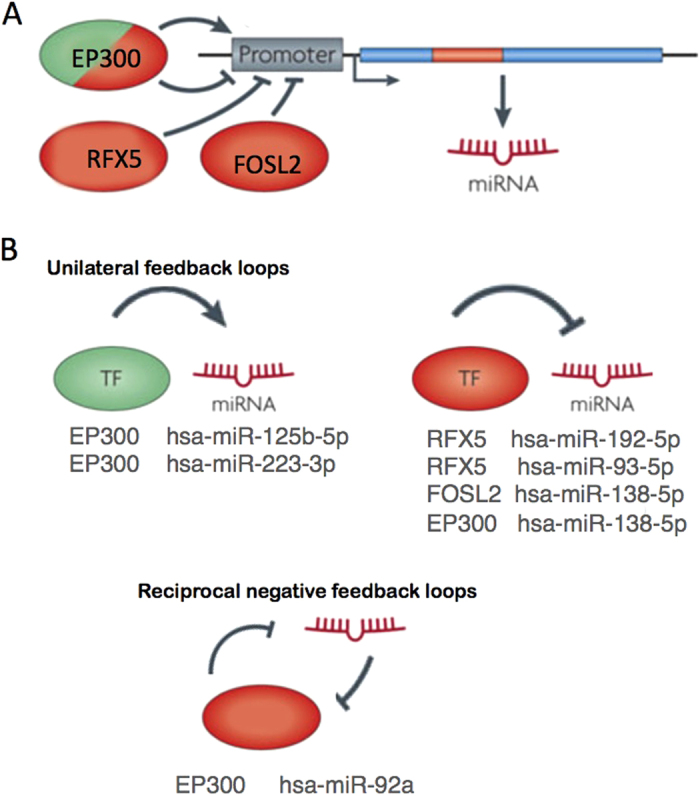
Activators and repressors of the 44 enriched miRNAs. When screening the negatively correlated target genes of the 44 enriched miRNAs, we found three TFs able to up- or downregulate miRNA expression (*EP300*, *RFX5* and *FOSL2*). The promoter regions of the expressed miRNAs are very similar to those of protein-coding genes. The presence of CpG islands, TATA box sequences, initiation elements and certain histone modifications indicate that the promoters of miRNA genes might be controlled by TFs. (**A**) *RFX5* and *FOSL2* both might inhibit expression of miR-192-5p, miR-93-5p and miR-138-5p in blood, although *EP300* might stimulate expression of miRNAs such as miR-125-5p and miR-223-3p. (**B**) Unilateral or reciprocal-negative feedback loops result in oscillatory or stable mutually exclusive expressions of the TFs and miRNA components^23^. For instance, *EP300* might repress miR-92a expression. miR-92a targets the *EP300* TF and might block its expression. Figure adapted from Krol *et al.*^23^.

Furthermore, we found that the regulatory network based on the 351 target genes included 31 TFs (Supplementary Table S10). Our results suggest that the *EP300, RFX5* and *FOSL2* TFs regulated the expression of six different miRNAs within the regulatory network (Fig. 7). For example, the EP300 protein stimulated transcription of miR-92a, which in turn suppressed *EP300* expression. More sophisticated regulation was provided by dual negative-feedback loops, such as the one involving miR-138-5p and the TFs *FOSL2* and *EP300* (Fig. 7).

### Validation of the enriched miRNAs and their inversely correlated target DEGs by applying graphical Gaussian models (GGMs) to a validation set

**Figure 8:**
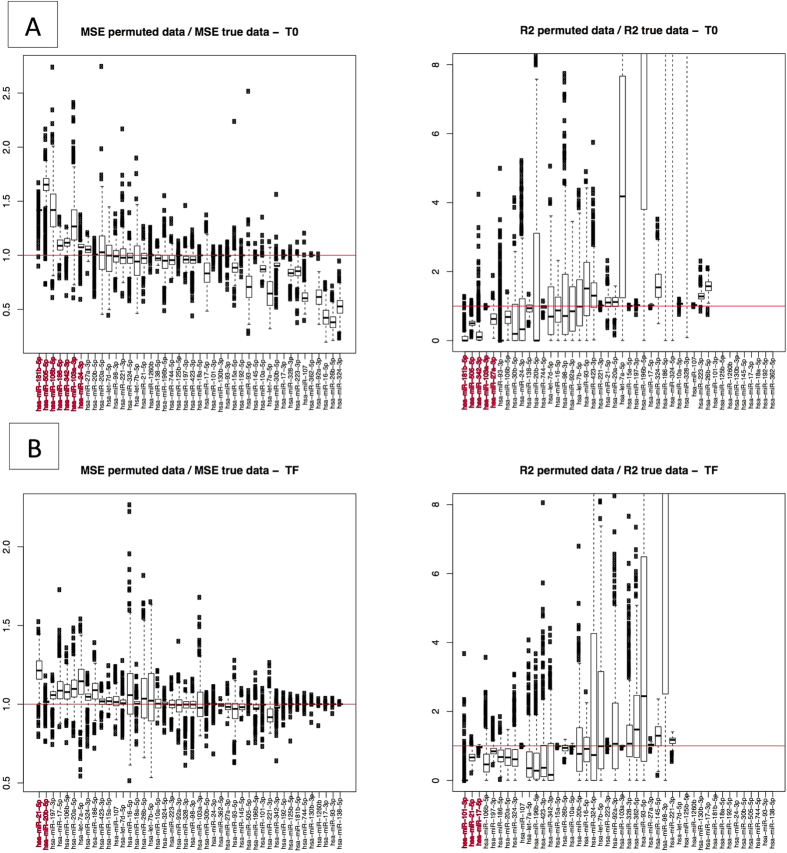
Validation of the 44 enriched miRNAs by applying GGMs to an independent validation set. For each selected enriched miRNA, MSE and R^2^ were computed through a GGM implemented on the 44 enriched miRNAs and their 351 inversely correlated target genes. (**A**) The upper row of boxplots shows the ratio between MSE values for the randomly selected genes from among the 24,415 genes and candidate gene set at T0, and the values of the R^2^ for predicted vs. observed values at T0, respectively. (**B**) The lower row of boxplots shows the same boxplots at T1. In all cases, a total of 500 repetitions were performed with 351 genes randomly selected from among the 24,415 genes for each miRNA. For both criteria, we computed permutation *p*-values, i.e. the proportion of repetitions leading to a smaller MSE (or a larger R^2^) with respect to the values found with the originally selected 351 genes. MicroRNAs with *p* < 0.10 are shown in red.

We next used an independent cohort of animals to validate the inversely correlated target-miRNA regulatory network by applying GGMs in a regression framework. This cohort included 16 horses sampled solely at T0 and 31 other horses sampled solely at T1. The models were built with *p* = 44 miRNAs and *j* = 351 inversely correlated DEGs targeted by at least one of these miRNAs. In order to increase the degree of precision, we specified a list of edges constrained to zero: only (i) interactions between miRNAs and their experimentally validated target genes, and (ii) gene-gene interactions predicted by PPI were considered (Supplementary Fig. S3). For each time point (T0 and T1) and each enriched DEmiRNA, the coefficient of determination (R^2^) and the mean squared error (MSE) were computed and compared with the values obtained with 351 repeatedly randomly selected genes (n = 500 times). Moreover, for each criterion, the extent to which each DEmiRNA was predicted by its inversely correlated target genes (as a *p*-value) was calculated as the proportion of repetitions with more statistically significant results than the original data. None of the enriched DEmiRNAs had an adjusted *p*-value of below 0.05 in the FDR test (Supplementary Table S11). Although the adjusted *p*-values were not statistically significant, these analyses allowed us to rank the enriched DEmiRNAs according to the strength of the direct interaction with their inversely correlated target genes (Fig. 8). At T0, the MSE and R^2^ criteria were concordant, displaying *p-*values < 0.05 for miR-181b-5p and miR-505-5p. At T1, miR-21-5p displayed a *p*-value < 0.05 for both R^2^ and MSE; this miRNA was not predicted by either R^2^ or MSE at T0, suggesting that it might be involved in the regulation of exercise-related physiological processes (Fig. 8).

## Discussion

Endurance exercise has a profound impact on metabolism in tissues other than skeletal muscle (including heart, brain, adipose tissue and liver)^25^. The level of exercise performed by horses during an endurance competition is similar to that of a human marathon runner^26^ or ultramarathon runner^27^. By using microarrays to analyse the blood transcriptome and miRNome, our objective was to identify and characterize candidate miRNAs and the interplay that ultimately resulted in the coordinated response to endurance exercise.

Peripheral blood is now widely used as a surrogate tissue for monitoring whole-body status^28^. Indeed, blood transcriptome analysis is undoubtedly capable of evidencing responses to exercise in the horse^26^,^29^,^30^. By comparing the respective blood transcriptome profiles before and after exercise, we were able to identify a large number of DEGs that fit neatly into the well-characterized context of adaptive regulation to exercise in endurance horses (including energy metabolism, the inflammatory response, stress resistance, oxidative stress, cell death and proteolysis^26^,^29^,^31^). These changes may help to supply the working muscles with energy or control excessive inflammatory reactions. They may also be involved in “staleness” and the transient immunosuppression that can occur during and/or after endurance exercise.

We found a total of 167 DEmiRNAs when comparing T1 with T0. These included two mitomiRs (i.e. DEmiRNAs encoded by the mtDNA), which might belong to the new family of miRNAs recently discovered in human vascular epithelium cells and matured from the mtDNA-transcribed long, non-coding RNA^32^. Endurance exercise is the most potent physiological inducer of mitochondrial biogenesis in skeletal muscle^33^. We hypothesize that the increase in mitochondrial biogenesis and cell energy regulation in endurance exercise is boosted by these mitomiRs. Despite the relatively small number of animals studied, these findings are valuable.

In order to assess the main effects of DEmiRNA regulation on exercise transcriptome patterns, we used the hypergeometric test and its generalization to search for DEmiRNAs with significant target site enrichment within DEGs. In fact, 44 DEmiRNAs of the 167 DEmiRNAs had significantly more targets among DEGs than among the transcriptome as a whole. Twenty-one of these 44 enriched miRNAs were already known to be related to exercise adaptation in humans, whereas all the others were identified for the first time in the present study. Hence, our results have added to the list of miRNAs expressed in blood following exercise. Future studies will have to determine whether this novel list of orthologous equine miRNAs expressed in blood is horse-specific or reflects a more general response to exercise in mammals (e.g. with counterparts in humans). More precisely, the observed differential expression of miR-15a, miR-16, miR-17, miR-18a, miR-20ab, miR-21-5p, miR-27a, miR-30b, miR-93, miR-101, miR-106, miR-107, miR-125b, miR-130, miR-138-5p, miR-145, miR-181ab-5p, miR-221, miR-223, miR-342-3p and miR-505 was consistent with previously reports on exhaustive exercise in humans^14^,^13^,^20^,^21^,^34^. Many of the enriched DEmiRNAs observed in our study are known to originate from blood cells and to regulate genes that are involved in immune processes, apoptosis and transcription regulation. For example, miR-21, miR-27a and miR-181 were found to be DE in the whole blood of highly trained human athletes after a 30-minute treadmill test^22^. It was then suggested that these miRNAs regulate physiological processes that are essential for the response to exercise (such as immune function, apoptosis, membrane traffic of proteins and transcriptional regulation)^22^. Furthermore, miR-17, miR-18a, miR-20a (all members of the miR-17-92 cluster), miR-106 and miR-93 (paralogs of the miR-17-92 cluster) were found to be DE in circulating neutrophils of 11 men after a brief bout of exercise; the miRNAs regulated the ubiquitin-mediated proteolysis pathway^21^. Lastly, we observed differential expression of miR-181, which is known to be affected in different types of leukocyte^35^ and likely to be involved in the regulation of some fundamental adaptive changes in the immune system during endurance exercise^34^. Similarly, Makarova *et al.*^34^ suggested that the exercise-induced expression of miRNA-181 in blood cells may be a compensatory, anti-inflammatory adaptation to the primary, systemic inflammatory response caused by exercise.

The ability to exercise for extended periods requires not only adequate endocrine and immune machinery but also energy sources^3^. Studies on the role of miRNAs in these adaptive processes have just started. Some preliminary results in humans suggest that the post-exercise increase in miR-107 expression regulates glucose homeostasis in liver and adipose tissue^15^, whereas in the horse the increased expression of miR-17 after exercise might be linked to the regulation of glucose metabolism in different tissues^30^. Investigation of the adaptive significance of each individual miRNA expressed in blood is still in its infancy, and the function of many miRNAs remains unknown.

The earliest studies in this field also have demonstrated that endurance exercise-induced changes in plasma/serum levels of circulating miRNAs (c-miRNAs)^13^,^15^,^16^,^17^,^18^,^19^. In most reports, plasma levels of human miR-1^36^, miR-20^13^,^18^, miR-21^13^,^18^, miR-103^15^, miR-107^15^, miR-126^19^, miRNA-133^19^,^36^, miR-146a^13^,^18^, miR-206^36^, miR-221^13^,^18^ and miR-222^13^,^18^ were found to be higher after endurance exercise – suggesting a possible role in exercise adaptations. Similarly, we observed an enrichment of miR-20ab, miR-21, miR-103a-3p, miR-107 and miR-221 when comparing pre- and post-ride blood samples. However, it is probable that most of the miRNAs in the present study came from blood cells, since the microarray-based estimation of circulating miRNA abundance from total blood RNA levels is particularly difficult^37^. In the present study, the c-miRNAs may have been partly masked (or at least diluted) by greater amounts of cellular miRNAs^38^. Furthermore, we only considered miRNAs that displayed a signal intensity greater than the mean +1.5 SD for the negative control; as a result, we probably missed some potentially detectable c-miRNAs. Baggish *et al.*^13^ suggested that human miR-20a, miR-21 and miR-221 (i) are released after exercise into the bloodstream by tissues other than blood cells and (ii) may regulate key pathways in angiogenesis, inflammation, muscle contractibility and adaptation to hypoxia. However, we believe that blood cells may have significantly contributed to our present results. Accordingly, levels of miR-20a, miR-21 and miR-221 were not correlated with changes in plasma markers of muscle inflammation and liver damage. In contrast, however, we found some significant, positive correlations between miR-133 levels and changes in plasma creatine kinase and aspartate transaminase levels (which reportedly reflect cell damage in human athletes after a marathon race^19^). In view of this result, the relationship between blood cell levels and plasma/serum levels of miR-133 also warrant further examination.

A further observation of interest relates to the fact that the 44 enriched miRNAs regulated a total of 351 inversely correlated target DEGs. All target genes were involved in processes highly relevant to the response to exercise, including immune function, apoptosis, protein degradation, transcription regulation, and mitochondrial biogenesis and energy metabolism. We hypothesized that these 351 target genes were driven not only by the miRNAs within the regulatory network but also by some of the TFs. As further confirmation of this hypothesis, we found that the regulatory network based on the 351 target genes and 44 enriched miRNAs featured a high proportion of TFs (n = 31) – including *PPARγ*, one of the master regulators of mitochondrial biogenesis and energy metabolism^39^. Furthermore, the assembly of miRNAs and TF co-regulators within the regulatory network revealed several interesting feed-forward and feedback loops.

After we had examined the structural and functional aspects of the co-regulatory networks, we further confirmed the regulatory effects of the 44 enriched miRNAs (identified by computational predictions on an experimental set of 14 animals) using an independent cohort of animals and a GMM approach. Our data confirmed a seldom-measured relationship between target DEGs and their miRNA regulators. Interestingly, we confirmed the post-exercise expression of miR-21-5p and their target DEGs. The expression of miR-21-5p is known to be stress-responsive; this miRNA has an important role in heart failure^40^, renal ischemia reperfusion injury^41^, and in the self-protective anti-inflammatory reaction to exercise^22^. Aforesaid, the up-regulation of circulating miR-21-5p in humans was reported to occur in plasma of endurance athletes^13^,^14^,^15^,^16^,^17^,^18^ and in circulating PBMCs^20^ and blood cells^22^ upon exercise. At baseline (T0), we confirmed the regulatory roles of miR-181a-5p, miR-505-5p and their target DEGs. As mentioned above, miR-181 in PBMCs has already been associated with a T-cell response to exercise in humans^20^, and Makarova *et al.*^34^ have suggested that miR-181 has several key roles in the adaptation to exercise. In contrast, miR-505-5p has not previously been linked to the regulation of exercise.

Our study had several limitations. Firstly, our main analysis was based on human orthologous miRNAs, for which we had a comprehensive list of validated miRNA–target interactions. We therefore had to ignore the predicted miRNA–target interactions that were specific to the horse. Moreover, the identification of putative equine miRNAs using sRNAseq was performed using samples derived from six tissues other than blood and with small number of biological replicates; this may have prevented us from detecting certain blood-specific or exercise-induced miRNAs. Secondly, there are several modes of gene regulation other than down-regulation by miRNAs: these include up-regulation by miRNAs, DNA methylation and chromatin remodelling—all of which may be involved in the responses to exercise. Furthermore, chemical perturbations (such as changes in pH and local temperature), systemic increases in cytokine and growth factor levels, and other factors such as the exercise dose, gender, age, genetic background and stochastic factors can all potentially modify transcriptome expression. Therefore, future research will have to focus on how these various regulatory interactions are synchronized to maintain homeostasis during and following endurance exercise in horses. Thirdly, we analysed miRNAs from whole blood. Thus, it remains to be determined whether the individual components of this heterogeneous fluid (i.e. plasma, platelets, erythrocytes, nucleated blood cells and exosomes) reflect the overall microRNA response to exercise in horses. With regard to the requirement for highly specific c-miRNA biomarkers of metabolic demand, muscle damage, myocardial injury or endotoxaemia during equine endurance events, it may make sense to focus on c-miRNAs not expressed by blood cells. However, it must be borne in mind that intravascular haemolysis occurs very frequently during and after intense exercise in horses^42^,^43^, which can significantly affect levels of circulating miRNAs

Despite these limitations, our results extend earlier observations on gene expression in equine athletes^26^,^29^, and we showed that the main changes in the blood transcriptome appear to be driven by changes in the expression of *trans*-acting regulators in general and 44 enriched DEmiRNAs in particular. Characterization of these miRNAs that lead to endurance-exercise-mediated systemic adaptation might also lead to the development of novel nutritional, pharmacological, and exercise-based training interventions. The goal would be to fine-tune levels of miRNA expression and thus improve the athlete’s energy metabolism and inflammatory response. Moreover, our results also demonstrated the sensitivity and specificity of whole-blood derived mRNA and miRNAs as markers for the response to endurance exercise. In view of the availability of whole blood sample tubes that protect the RNA content and the minimal handling required during sample collection, the use of whole blood has some clear advantages for monitoring the post-exercise expression of miRNAs under field conditions. Lastly, the specialized nature of equine musculoskeletal tissues (effective high-speed locomotion and strength), the large size of both human and equine species, the good degree of access to tissue specimens, and the high overall degree of similarity between equine and human blood transcriptome responses to exercise make the horse a good natural model for basic exercise research.

## Conclusions

The microRNA and gene co-regulatory network profiled in blood reflects the physiological regulation of the transcriptome following endurance exercise in the horse. Our present results suggest that 44 enriched miRNAs and their 351 inversely correlated target genes regulate lipid metabolism, carbohydrate metabolism, mitochondrial biogenesis, proteolysis and the immune response. By using various computational methods and an independent cohort of animals, we confirmed that miR-21-5p, miR-181b-5p and miR-505-5p are putative regulators of the response to endurance exercise. Our results also suggest that (i) a variety of mechanisms underlie adaptive changes to the transcriptome and (ii) changes in the expression of a few key regulators may improve the metabolic and inflammatory response to exercise in athletes.

## Methods

### Animals

Sixty-one pure-breed or half-breed Arabian horses (20 females and 41 geldings; mean ± SEM age: 10 ± 2) competing in three 160 km endurance competitions were recruited with the owner’s agreement. The weather conditions, terrain difficulty and altitude for the three endurance competitions were similar. The 61 horses were checked carefully for the population-genetic structure and covariates such as age, gender, and environmental influences (disease states, medical treatment, etc.). To ensure sample homogeneity, the participating horses were subject to the same management practices throughout the ride and passed the International Equestrian Federation (FEI)’s compulsory examination before the start. Animals were fed 2–3 hours before the start of the endurance competition with *ad libitum* hay and 1 kg of concentrate pellets. During the endurance competition, all the animals underwent veterinary checks every 20 to 40 km, followed by recovery periods of 40 to 50 minutes (in accordance with the FEI rules on endurance riding). After each vet gate check, animals were provided with *ad libitum* water and hay and a small amount of concentrate pellets. The median winning speed over the entire ride was 15.7 ± 1.04 km/h. Information on training, nutrition and medical examinations was obtained but it was impossible to control for the feed composition and levels of consumption. The study design was approved by the independent ethics committee at Alfort Veterinary School and the University of Paris Est (reference: 12/07/11-1). All methods were carried out in accordance with the approved guidelines. In all cases, the owner provided his/her informed consent prior to the initiation of study procedures with the animals. Further details have been published by Le Moyec *et al.*^44^.

The horses were divided into two non-overlapping sets: the experimental set and the validation set. The validation set came from an independent cohort of animals, to ensure that the observed effects were reproducible in a broader context. The experimental set included 14 horses sampled before the 160 km ride (T0, baseline) and then again afterwards (T1). Samples were collected within 30 minutes of the end of the ride. Two horses in the experimental set failed a vet gate check (poor metabolic condition after 80 km and lameness at the end of the ride, respectively).

The validation set included 16 horses sampled solely at T0 and 31 other horses sampled solely at T1. Of the horses sampled at T1, four were eliminated for lameness after 106 km (n = 1), 133 km (n = 2) or 160 km (n = 1), two were eliminated after 30 km for metabolic disorders and one was eliminated for tiredness after 130 km.

### Blood biochemical assays

Blood samples were collected in dry tubes at the end of the endurance event. After clotting, the tubes were centrifuged and the harvested serum was stored at 4 °C until analysis (no more than 48 later, in all cases). Sera were assayed for total bilirubin, conjugated bilirubin, total protein, creatinine, creatine kinase, aspartate transaminase, gamma glutamyltransferase and serum amyloid A levels on a RX Imola analyser (Randox, UK). Blood collected in EDTA tubes was used to measure the packed cell volume after centrifugation.

### Blood samples, RNA isolation and microarray experiments

A single blood sample for both transcriptome and miRNome profiling was obtained in order to study direct relationships between mRNA expression and miRNA expression in each animal. Whole blood samples from each horse were collected in PAXgene Blood RNA tubes (Qiagen, Germany) and the total RNA was isolated using a PAXgene Blood RNA Kit (Qiagen), according to the manufacturer’s instructions.

The RNA’s purity and concentration were determined using a NanoDrop ND-1000 spectrophotometer (Thermo Scientific, USA) and RNA integrity was assessed using the Bioanalyzer 2100 (Agilent Technologies, USA). Each of the 61 RNA samples was then divided into two aliquots for use in the miRNA microarray or gene expression microarray experiments, respectively.

The transcriptome was profiled with a custom equine 4 × 44 K microarray (ID: 044466, Agilent Technologies). Cyanine-3 (Cy3)-labelled cRNAs were prepared from 100 ng of total RNA using the One-Color Low Input Quick Amp Labeling kit (Agilent Technologies). Specific activities and cRNA yields were measured using the NanoDrop ND-1000 (Thermo Scientific, USA). For each sample, 1.65 μg of Cy3-labeled cRNA (specific activity >9.0 pmol Cy3/μg of cRNA) were fragmented at 60 °C for 30 minutes in a volume of 55 μl containing 25× Agilent Fragmentation Buffer and 10× Agilent Blocking Agent. Subsequently, 55 μl of 2× Agilent Hybridization Buffer were added to the fragmentation mixture and hybridized to the array, in accordance with the recommended protocol. After hybridization, the microarrays were washed 1 minute at room temperature with GE Wash Buffer 1 (Agilent Technologies) and 1 minute at 37 °C using GE Wash Buffer 2 (Agilent Technologies).

The slides were then scanned using a G2565CA Scanner System (Agilent Technologies), using a scan protocol with a resolution of 3 μm and a dynamic range of 20 bits. The resulting TIFF images were analysed with the Feature Extraction Software v10.7.3.1 (Agilent Technologies), using the GE1_107_Sep09 protocol. The microarray data can be obtained from the Gene Expression Omnibus (GEO) database^45^ with the accession number GSE72973 (http://www.ncbi.nlm.nih.gov/geo/query/acc.cgi?acc = GSE72973).

For the miRNome profiling, an Agilent custom equine miRNA 8 × 60 K microarray was used (ID: 060464). For each sample (n = 61), a total amount of 100 ng of total RNA was dephosphorylated, 3′ end-labelled with Cy3-pCp, purified on Micro Bio-Spin 6 columns (Bio-Rad, USA), dried, and hybridized to the arrays using the miRNA Complete Labeling and Hybridization Kit (Agilent Technologies) according to the manufacturer’s instructions. After a washing step, hybridized microarray slides were scanned using a G2565CA Scanner System (Agilent Technologies) and a scan protocol with a resolution of 3 μm and a dynamic range of 20 bits. The resulting TIFF images were analysed with the Feature Extraction Software v10.7.3.1 (Agilent Technologies), using the miRNA_107_Sep09 protocol. The microarray data are available at GEO (GSE73102: http://www.ncbi.nlm.nih.gov/geo/query/acc.cgi?acc = GSE73102). The whole reference series (mRNA and miRNA) is available at GEO (GSE73104: http://www.ncbi.nlm.nih.gov/geo/query/acc.cgi?acc = GSE73104).

Microarray dataset was deposited in GEO, in accordance with the MIAME guidelines.

### Description of the Agilent custom equine miRNA 8 × 60 K microarray

The custom equine array contained 1,886 *Homo sapiens* (*hsa*) miRNA sequences from the miRBase database (V9.2). *Hsa* miRNAs were included in the custom equine microarray because Buza *et al.*^46^ reported that over 60% of known mature equine miRNAs had perfect matches with human-associated miRNAs. Furthermore, the microarray included 84 known equine (eca) miRNAs (with no known human homologs), 876 putative novel miRNAs and 20 putative small RNAs encoded by the mitochondrial DNA. The putative novel sequences were identified in an independent sRNAseq experiment during which we used 16 small RNA libraries from six different equine tissues (heart, liver, cartilage and three muscles: the platysma, the *gluteus medius* and the masseter). miRNA from the platysma and masseter muscles, heart and liver were extracted from 15 healthy horses of different ages and breeds (collected at the slaughterhouse). MicroRNAs from the *gluteus medius* were extracted from 10 healthy and 8 Cob Normand horses affected by polysaccharide storage myopathy. Total RNA was purified from tissues using the miRNeasy Mini kit (Qiagen) and the RNeasy MinElute Cleanup kit (Qiagen) for enrichment of small RNA (<200 bp), according to the manufacturer’s protocol. Total RNA quality and concentration were assessed as described above. For each tissue, three pools of three horses were formed (i.e. yielding 16 libraries). Approximately 500 ng of total RNA were used for library constructions, according to the manufacturer’s protocol for the SOLiD® Total RNA-Seq Kit (Applied Biosystems, USA). The small RNA libraries were sequenced on a SOLiD 5500XL Series Genetic Analysis System at the Metaquant core facility (INRA, Jouy-en-Josas, France). Solid sequencing generated a total of 350 million reads of ~50 bp. The overall flow of the sequencing and bioinformatics analysis for small RNA has been described previously^47^. In brief, after removal of low-quality reads, the flanking linker and primer sequences, all remaining small RNAs were aligned with the *Equus caballus* genome (Equ Cab 2; GCA_000002305.1) with BowTie^48^ (v0.12.7). Fewer than 2 mismatches were required. Reads with >6 alignments were removed. Mapped reads overlapping with known non-coding RNAs obtained from RFAM (http://rfam.sanger.ac.uk/) were also discarded. To detect putative miRNAs within the small RNAs, reads were filtered by length (17–27 bp). Sequences that mapped solely to the mitochondrion were designed as mitomiRs. Subsequently, all potential novel miRNAs and mitomiRs were sorted and assigned to the putative ncRNA locus (i.e. clusters). Clusters with fewer than 10 reads were discarded because of their low information content. Potential pre-miRNAs were excised. Excision was initiated when a stack of reads was encountered. If there was a higher read stack within 20 bp downstream of the current read stack, the former stack was selected. Hence, the highest local read stack was identified. Next, sequences covered by the highest local read stack were excised twice: once including the 50 bp upstream and 10 bp downstream flanking sequences, and once including 10 bp upstream and 50 bp downstream flanking sequences (to simulate the 5p and 3p positions of the potential miRNA). These putative pre-miRNAs were then screened for microRNA-like hairpin structures with RNAfold (using the latter’s default settings). For further details on sequencing analysis and prediction of novel miRNAs, see Desjardins *et al.*^47^. Our analysis yielded a total of 876 novel miRNAs and 20 putative mitomiRs (Supplementary Table S12). Three novel miRNAs were re-annotated as eca-miRNAs because they have been already included in the new update of the miRNA database (with new-eca-mir-824 updated to eca-miR-1388; new-eca-mir-1047 to eca-miR-676; and new-eca-mir-1072 to eca-miR-2114). Among the plausible mitomiRs, two mapped to the 16 S rRNA, nine mapped to various tRNAs, two mapped to genes for complex I subunits, two mapped to genes from complex IV subunits, and two mapped to genes from complex V subunits. Lastly, two potential mitoRNAs mapped to the D-loop locus (Supplementary Fig. S4). None of the predicted pre-miRNA sequences encoded by the mitochondrial genome were significantly folded into a duplex structure.

### Statistical analysis of the microarray results

All the transcriptome pre-processing, normalization and statistical analysis steps were carried out using several Bioconductor packages in R programming language (version 3.02). Firstly, the data’s quality was checked with the arrayQualityMetrics package^49^. Subsequently, probes showing any of the following Agilent flags (gIsFeatPopnOL = 1, gIsBGNonUnifOL = 1, gIsFeatNonUnifOL = 1) were removed from the analysis. The raw intensity values were then background-corrected using the “normexp” function, and the expression data were quantile-normalized (using the Limma package^50^; version 3.14.4). Probe summarization was based on the median expression value for the replicated probes. A total of 24,415 genes were selected in this analysis. Given that many of the original annotations for the custom equine Agilent microarray (AMAMID 021322, enriched with 384 equine transcripts) have been found to be incomplete, the annotation available in June 2014 at (http://www.genomics.agilent.com/en/Custom-Design-Tools/eArray/?cid=AG-PT-122&tabId=AG-PR-1047) was used to re-assign the probes to new probe sets.

PCA was performed with FactoMineR and Ade4 packages (version 1.23), in order to establish whether a particular array contributed markedly to variability in the gene expression data (i.e. whether it retained most of the information). Differences revealed by the PCA were assessed using the Monte Carlo Permutation Procedure (999 replicates; “randtest” function) in the Ade4 package in R. DEGs were identified by using the Limma package. The statistical linear model included the time point (T0/T1) as a fixed effect. To make the analysis more robust and control more strictly for the false discovery rate (FDR), the *p*-values were corrected for multiple testing using Bonferroni’s method with a threshold of adjusted p < 0.05^51^. An unsupervised analysis of DEGs was carried out to identify clusters of samples or genes on the basis of their variance-covariance structure. Thus, a two-way hierarchical cluster analysis was performed using the *hclust* function with “1-cor (x)” as the distance and different aggregation criteria. The *heatmap* function was used to generate images.

For miRNome profiling, the raw data was pre-processed using a variant of the robust multi-array average (RMA) algorithm that has been specifically implemented for Agilent miRNA microarrays by Lopez-Romero *et al.*^52^. The RMA implementation in the *AgiMicroRna* package was used without the initial background correction step, as recommended^52^. A miRNA transcript was considered to be detectable only if it met the following two conditions: (i) expression in at least 33% of the experimental samples; (ii) a signal intensity above the mean value of the negative control +1.5 standard deviations. DEmiRNAs were identified using the Limma package. In this case, correction for multiple testing (FDR < 0.05) was performed using the Benjamini-Hochberg method (as a compromise between the unadjusted analysis and the Bonferroni procedures). Multivariate analysis was performed as explained above.

### Generation of a functional annotation map from a list of genes

The GO term and the Kyoto Encyclopaedia of Genes and Genomes (KEGG) enrichment analyses of DEGs and target DEGs were performed using Cytoscape V2.7 (http://cytoscape.org/) with the ClueGo V1.3 plug-in^53^. ClueGO determines the distribution of the gene list for the various GO terms and pathways. The *p*-value was calculated using right-sided hypergeometric tests and the Benjamini-Hochberg correction for multiple testing (FDR < 0.001). Together with this very stringent FDR threshold, a high kappa value (0.4) enabled us to precisely select GO terms enriched in highly connected genes. The size of the nodes reflected the term’s degree of enrichment. The network was automatically laid out using the organic layout algorithm in Cytoscape. Functional groups were created by iterative merging of the initially defined groups, according to the predefined kappa threshold. Only functional groups represented by their most significant term were visualized in the network^53^. As a complementary approach, the list of selected genes was also fed into an Ingenuity Pathway Analysis (IPA; version 5.5, Ingenuity Systems, USA) to identify relevant categories of molecular functions, cellular components and biological processes. The IPA enabled us to identify (i) significantly overrepresented functional GO annotations, (ii) their over- or under-expression, and (iii) group-specific transcriptional networks. All listed or reconstructed cellular pathways were derived from the expert-annotated Ingenuity Knowledge Base of over 10^6^ PPIs. The IPA output a statistical assessment (based on Fisher’s exact test) of the significance of representation for biological functions and signalling pathways. The IPA computed networks and ranked them according to a statistical likelihood approach.

### Use of iRegulon to identify TFs involved in biological processes

The iRegulon computational method was used to identify TFs within the set of DEGs and target DEGs^54^. iRegulon searches the regulatory sequences around each gene in order to detect enriched TF motifs. It uses a database of nearly 10,000 TF motifs. iRegulon links enriched motifs to candidate TFs and determines the optimal subset of direct target genes.

### DEmiRNAs and their inversely correlated target DEGs: enrichment analysis

For each DEmiRNA, we assembled a comprehensive list of all experimentally validated target genes (using the multiMiR package in R). multiMiR is a comprehensive collection of predicted and validated miRNA–target interactions and their associations with diseases and drugs^55^. It contains human and murine data from 14 external databases that are categorized into three components, including three validated miRNA–target databases (miRecords, miRTarBase and TarBase), eight predicted miRNA–target databases (DIANA-microT, ElMMo, MicroCosm, miRanda, miRDB, PicTar, PITA and TargetScan), and three disease- or drug-related miRNA databases (miR2Disease, Pharmaco-miR and PhenomiR)^55^.

To investigate the biological relevance of the DEmiRNAs, we used two complementary statistical approaches. For each of the DEmiRNAs presenting at least one target gene, we performed: (i) the hypergeometric test for enrichment in targets DEGs negatively correlated with the DEmiRNA; and (ii) the generalization of the hypergeometric test. Both methods were implemented on the experimental set. For the hypergeometric test, we performed a pair-wise Pearson correlation analysis of the expression levels of DEGs and the expression levels of DEmiRNAs presenting at least one experimentally validated target. Subsequently, we subtracted the set of correlated DEmiRNAs-DEGs with r < −0.5 and *p* < 0.05. One should note the existence of two types of negative regulatory interactions: (i) over-expressed mRNA/under-expressed miRNA and (ii) under-expressed mRNA/over-expressed miRNA. We next looked at whether the DEmiRNA targets were over-represented among the subtracted set of genes, when compared with the other genes in the transcriptome. Benjamini and Hochberg correction^56^ for multiple testing was applied to the 91 *p*-values obtained.

Moreover, in order to address the hypergeometric test’s lack of power when the number of inversely correlated target genes for a given DEmiRNA was very small, we also implemented a variant of the hypergeometric test. In contrast to the hypergeometric test (which is focused on the number of genes that were both DEGs and inversely correlated with a DEmiRNA at fixed significance thresholds), the generalization of the hypergeometric test considers all possible thresholds. In this variant, we tested for enrichment in target genes by selecting DEGs regardless of the sign of their correlation with the miRNA. When considering a given DEmiRNA with at least one experimentally validated target gene, we looked at whether their target genes presented smaller *p*-values than the other genes in the transcriptome using a one-sided Wilcoxon rank sum test (implemented with the “wilcox.test ()” function in R). The FDR was determined to correct for multiple testing. Only the enriched DEmiRNAs for which at least one target gene was inversely correlated (r < −0.5) were selected. Lastly, only those DEmiRNAs that were significant either in the hypergeometric test or its generalization after correction for multiple testing (FDR < 0.1) were analyzed further. The FDR was set to 0.1 in order to retain as many biological functions as possible.

To find possible associations between the enriched DEmiRNA expression and biochemical blood parameters, a Pearson and a non-parametric Spearman rank correlation were applied using the cor () function in R.

### Construction of a PPI sub-network on the basis of miRNA-mRNA interactions

The latest available version of the human PPI datasets from BioGRID (http://thebiogrid.org/) (release 3.2.105) was used for our analysis. The curated PPI data (containing 15,352 unique proteins and 281,862 interactions) constituted the parental PPI network. The Cytoscape program was used to visualize and analyze PPI networks. Firstly, the BioGRID parent PPI network was imported into Cytoscape. The target DEGs (human orthologous gene symbols) were listed in a text file (down regulated and up regulated DEGs, respectively) and mapped to the parental PPI network using the command “Select R Nodes R From ID List File”. To confine the analysis to interactions close to the target DEGs and to achieve maximum significance, only first-level interactions between target DEGs and their neighbours were detected.

### Network topology analyses

The networks’ topology was analysed using the NetworkAnalyzer Cytoscape plugin^57^. NetworkAnalyzer provides insights into the organization and structure of complex networks formed by the interacting molecules. It computes a comprehensive set of topological parameters, such as network diameter, density, centralization, heterogeneity, and clustering coefficient, neighbourhood connectivity, average clustering coefficients and the distribution of node degrees^57^. The degree of a node corresponds to the number of its directly connected neighbours. Briefly, the distribution of node degree *P*(*k*) is defined as the number of nodes with a degree *k* of 0, 1, 2, and so on. The dependency pattern can be visualized by fitting a line to the node degree data. NetworkAnalyzer calculates the positive coordinate value for fitting the line with the power law curve of the form y = βx^a^. The R^2^ value is a statistical measure of the linearity of the curve fit and used to quantify the fit to the power line. When the fit is good, R^2^ is very close to 1.

### Use of GGMs to validate DEmiRNA-gene association networks in a validation set

GGMs can infer direct relationships between variables within a set of repeated observations and in the absence or presence of *a priori* knowledge^58^. In the GGM method, networks are represented as undirected graphs. Each node represents a gene or a miRNA, and an edge connects two nodes if they are partially correlated. In contrast to correlation analyses (which measure both direct and indirect interactions between pairs of variables), partial correlation analyses measure the strength of direct interaction only^58^. A direct relationship between two variables corresponds to a non-zero entry in the partial correlation matrix. In a large-dimension context in which the number of variables exceeds the sample size, regularisation methods are needed for the estimation procedure. Moreover, in order to increase the estimation’s precision, some interactions between variables may be neglected by setting some edges to zero. We considered animals at T0 and animals at T1 separately. For each scenario, GGMs were built to model interactions between enriched miRNAs and DEGs that were inversely correlated and were targeted by at least one of the enriched miRNAs. In the correlation matrix, the following edges were set to zero: (i) miRNA-miRNA, (ii) miRNA-mRNA when the mRNA was not a validated target of the miRNA, and (iii) mRNA-mRNA when the interaction was not found in the BioGRID human PPI dataset (Supplementary Fig. S3). The analyses were carried out with the R package glasso, which uses a lasso penalty for GGM estimation and allows a specified list of edges to be set to zero. The value of the constant in the lasso penalty (which has to be specified in the glasso package) was set to 0.03 by minimising the cross-validation MSE described below.

Furthermore, GGMs can be viewed as regression models of each variable with regard to all the other variables; regression coefficients are then expressed as a function of the partial correlation matrix. The regression coefficient of a variable *i* with regard to a variable *j* is equal to zero if and only if there is an edge between nodes *i* and *j*^59^. The GGM method represents a refinement of simple linear regression models of genes with regard to each miRNA because it takes account of mRNA-mRNA correlations. We made use of this property to build a cross-validation procedure and thus estimate the predictive ability of each miRNA from the list of target mRNAs. We considered two criteria: (i) the cross-validation MSE, which increases with the precision of the prediction, and (ii) the R^2^ for predicted vs observed values, which decreases as the linear correlation between predicted and observed values increases. For each miRNA, MSE and R^2^ were computed on the network composed by the selected enriched miRNAs and their inversely correlated target genes. These MSE and R^2^ values were then compared with those obtained using the same procedure and the same number of genes randomly selected from the transcriptome. More precisely, for 500 repetitions, the genes were randomly selected from among a total of 24,415. Lastly, we computed permutation *p*-values, i.e. the proportion of repetitions leading to a smaller MSE (or a larger R^2^) with respect to the values observed with the originally selected genes.

### RT-qPCR validation of the transcriptome and miRNome results

To validate the technical robustness of the transcriptome data generated in the microarray study, quantitative RT-qPCR was carried out on a subset of candidate genes (Supplementary Table S13). We selected (i) genes with significant differences in expression levels when comparing T1 and T0 (a dynamic range of at least log2 (ratio) > 0.485), and (ii) genes of biological interest (e.g. *IRF3, CASB5,*). Specific PCR primers for these genes were designed using ABI Primer Express software (version 3.0.1, Applied Biosystems) with melting temperatures of 58 °C to 60 °C and product lengths of between 100 and 150 bp. Reverse transcription of 500 ng of isolated total RNA was performed using the SuperScript® VILO™ cDNA Synthesis Kit (Invitrogen, France), according to the manufacturer’s protocol. Dilutions (1/100) were used for qPCR with ABsolute Blue qPCR SYBR Green ROX mix (ThermoFisher Scientific, USA) in an Eppendorf Mastercycler RealPlex 4 (Eppendorf, France). The diluted cDNA samples were mixed with 1 × SYBR Green Master Mix and the specific reverse and forward primers (final concentration: 300 nM) in a final volume of 25 μl. Cycling conditions were 95 °C for 15 min, then 40 cycles of 95 °C for 15 sec and 60 °C for 1 min. For each sample and each gene, qPCR runs were performed in triplicate (in accordance with the manufacturer’s protocol). In order to quantify and normalise the expression data, we used the ΔΔCt method and the geometric mean Ct value from the succinate dehydrogenase complex, subunit A (*SDHA*), and beta actin *(ACTB)* as the endogenous reference genes. The set of genes chosen for confirmation by RT-qPCR was analysed in 10 animals of the experimental set using a linear effect model, including group (T0 or T1) as a fixed effect. The threshold for statistical significance was set to *p* < 0.05.

For analysis of the miRNome, five candidate miRNAs were selected from the microarray dataset for validation in the miRCURY LNA™ Universal RT microRNA PCR system (Exiqon, Denmark), using the same RNA samples used in the microarray profiling. cDNAs were obtained with the Universal cDNA synthesis kit II (product #203301, Exiqon, Denmark). The primer sets and their product numbers are given in Supplementary Table S14. The qPCR reaction was performed according to the kit manufacturer’s instructions (ExiLENT SYBR® Green master mix, product #203403, Exiqon). The hsa-miR191 (#204306, Exiqon, Denmark)) was used as endogenous control. As for mRNA, the set of miRNAs chosen for confirmation by RT-qPCR was analysed in 10 animals of the experimental set using a linear effect model, including group (T0 or T1) as a fixed effect. The threshold for statistical significance was set to *p* < 0.05.

## Additional Information

**How to cite this article**: Mach, N. *et al.* Integrated mRNA and miRNA expression profiling in blood reveals candidate biomarkers associated with endurance exercise in the horse. *Sci. Rep.* **6**, 22932; doi: 10.1038/srep22932 (2016).

## Supplementary Material

Supplementary Information

Supplementary Table S1

Supplementary Table S2

Supplementary Table S3

Supplementary Table S4

Supplementary Table S5

Supplementary Table S6

Supplementary Table S7

Supplementary Table S8

Supplementary Table S9

Supplementary Table S10

Supplementary Table S11

Supplementary Table S12

Supplementary Table S13

Supplementary Table S14

Supplementary Table S15
