# Supplementary material for: Integrated mRNA and miRNA expression profiling in blood reveals candidate biomarkers associated with endurance exercise in the horse
Source: Sci Rep. 2016 Mar 10;6:22932. doi: 10.1038/srep22932 (PMC4785432; doi:10.1038/srep22932)
Supplement: Supplementary Information [file srep22932-s1.doc]

**SUPPLEMENTARY INFORMATION**

**Integrated mRNA and miRNA expression profiling in blood reveals candidate biomarkers associated with endurance exercise in the horse**

Núria Mach *^1^, Sandra Plancade ^2^, Alicja Pacholewska^3^, Jerôme Lecardonnel^1^, Julie Rivière^1^, Marco Moroldo^1^, Anne Vaiman^1^, Caroline Morgenthaler^1^, Marine Beinat^1^, Alizée Nevot^1^, Céline Robert^1,4^ and Eric Barrey^1,5^

^1^ Animal Genetics and Integrative Biology unit (GABI), INRA, AgroParis Tech, Université Paris-Saclay, 78350, Jouy-en-Josas, France

^2^INRA, MaIAGE, Jouy-en-Josas, France

^3^Swiss Institute of Equine Medicine/Institute of Genetics, University of Bern and Agroscope, Bern, Switzerland

^4^ Université Paris-Est, Ecole Vétérinaire d’Alfort, Maisons-Alfort, France

^5^ Unité de Biologie Intégrative et Adaptation à l’Exercise, UBIAE, EA7362, Université d’Evry Val d’Essonne, Evry, France

**Supplementary results**

**Characterization of the transcription factors (TFs) that regulate DEGs**

We used iRegulon to search for TFs that were significantly overrepresented within the DEGs. The main putative regulatory TF in the network of DEGs was *ZFP42*, followed by the cooperatively transcriptional cofactors *SPI1, FOXO3, IRF3* and *NRF1* (Fig. 4 and supplementary Fig. S5). These co-regulated TFs were involved in the immune and inflammatory response, apoptosis, the response to stimuli, protein metabolism and even oxidative stress (supplementary Fig. S6). Using the TSmiR database [^1^](#_ENREF_1) (interaction maps on TFs and tissue-specific miRNAs derived from both experimentally validated and predicted data), we found that these TFs might control the spatiotemporal expression patterns of some of the DEmiRNAs (Fig. 4B). On one hand, mir-186-5p, miR-10a-5p, miR-223-3p and miR-205-5p might be induced by *SPI1* TF, whereas miR-192-5p might be inhibited by *IRF3* TF. On the other hand, miR-221-3p, miR-197-3p, and miR-744 might directly base-paired with *FOXO3* and repress or induce its expression (Fig. 4B). The combinatorial expression patterns for TFs, miRNAs and targets are shown in supplementary Fig. S5.

**Validation of mRNA and miRNA expression**

To further substantiate our findings, a subset of DEGs and DEmiRNAs was selected for validation with RT-qPCR. The RT-qPCR results confirmed our findings in most cases (supplementary Fig. S7).

**The PPI sub-network of the 44 enriched miRNAs and their depleted target DEGs**

In order to gain insights into how the target genes of the enriched miRNAs affected cellular biological activity, a full PPI screen of the 351 depleted target DEGs and their corresponding miRNAs was performed using the Reactome FI plugin in Cytoscape. A total of 337 genes presented PPI information and, with their first neighbouring proteins, formed a PPI sub-network containing 6,024 nodes and 21,314 edges (supplementary Fig. S8). The distributions of node degree followed an approximate power law distribution (R^2^ = 0.83), suggesting that the PPI network was scale-free and that a few protein nodes acted as hubs with a large number of links to other protein nodes. Other topological parameters of these sub-networks (such as the clustering coefficient, network centralization, and network density) are shown in supplementary Table S15. We also searched for interactions between target DEGs by carefully examining the expression of the individual TFs regulating the DEG-DEG PPI network. The *PPARγ* gene was found to be enriched, with a top motif ranked 9^th^ (normalized enrichment scores (NES) = 3.24) in the sub-network. This TF controlled the transcription of several genes within the network involved in lipid and carbohydrate metabolism (e.g. carnitine palmitoyltransferase 1A *(CPT1A)* and phosphoenolpyruvate carboxykinase (*PEPCK*)*;* supplementary Fig. S9).

**Correlation between enriched DEmiRNAs and biochemical blood parameters**

By slightly modifying Sawada et al.’s procedure [^2^](#_ENREF_2), we analysed the correlations between changes in the 44 enriched DEmiRNA levels and endurance-exercise-related biochemical blood parameters (i.e. creatine kinase (CK), the packed cell volume (PCV), and levels of aspartate transaminase (ASAT), gamma glutamyltransferase (GGT) and serum amyloid A (SAA), total plasma proteins, creatinine, conjugated bilirubin and total bilirubin). The results are summarized in supplementary Fig. S10. We found strong, significant, positive correlations (r>0.6; p<0.05) between dehydration parameters (PCV, creatinine and total protein concentrations) and several enriched miRNAs. We only found some weak correlations between the changes in several whole-blood derived miRNAs and the changes in plasma indicators of muscle and liver damage. Hence, future studies should (i) determine whether these whole-blood derived miRNAs are potential biomarkers for skeletal muscle damage or for damage to other tissues (such as the liver) during endurance exercises, and therefore (ii) clarify the effects of endurance exercise on the biogenesis and origin of these miRNAs.

Interestingly, when analysing the correlations for miR-133b and miR-133a-3p, we found some significant, positive correlations with plasma CK and ASAT levels (supplementary Fig. 11).

**Literature cited in the response the supplementary information section**

1 Guo, Z. *et al.* Genome-wide survey of tissue-specific microRNA and transcription factor regulatory networks in 12 tissues. *Sci Rep* **4**, 5150, doi:10.1038/srep05150 (2014).

2 Sawada, S. *et al.* Profiling of circulating microRNAs after a bout of acute resistance exercise in humans. *PloS one* **8**, e70823, doi:10.1371/journal.pone.0070823 (2013).

**Supplementary figures**

**Figure S1. Putative differentially expressed mitoRNAs when comparing pre- and post-ride samples**

A) A map of the horse mitochondrial genome, indicating the following features (from the centre, with the outer track corresponding to heavy strands and the inner track corresponding to light strands): GC skew+ (purple histogram), GC skew- (green histogram), GC content (black histogram), and start and stop codons. The positions of the putative mitoRNAs (small RNAs encoded solely by the mitochondrial DNA) are highlighted in red. B) Boxplots of the intensity distribution for the potential mitoRNAs (violet for T0 and orange for T1).

**
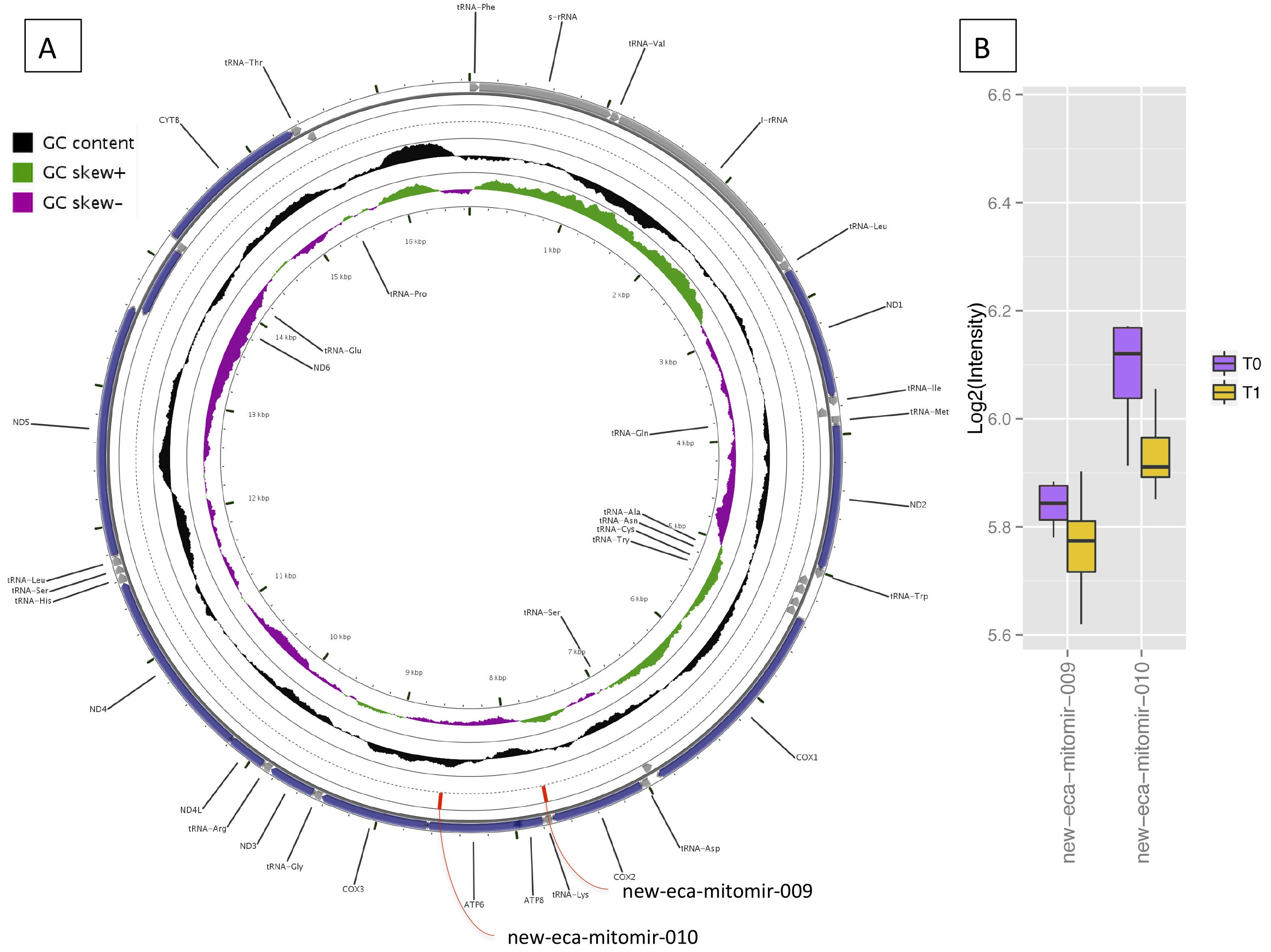
**

**Figure S2. Expression boxplot for the enriched miRNAs**

**
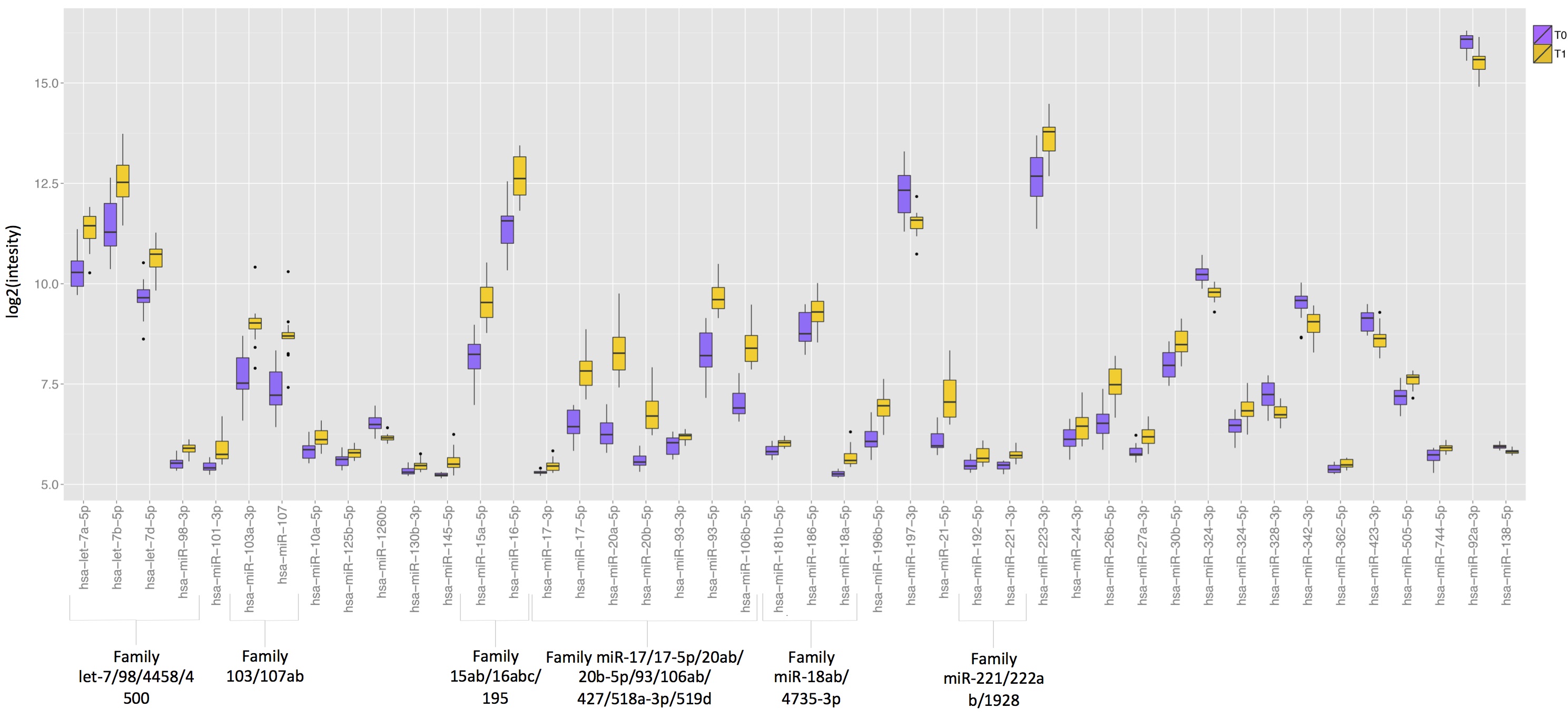
**

**Figure S3. GGMs: correlation matrix design**

In the correlation matrix, the following edges were set to zero: (i) miRNA-miRNA, (ii) miRNA-mRNA when the mRNA was not a validated-target of the miRNA, and (iii) mRNA-mRNA when the interaction was not reported in the BioGRID human PPI dataset.


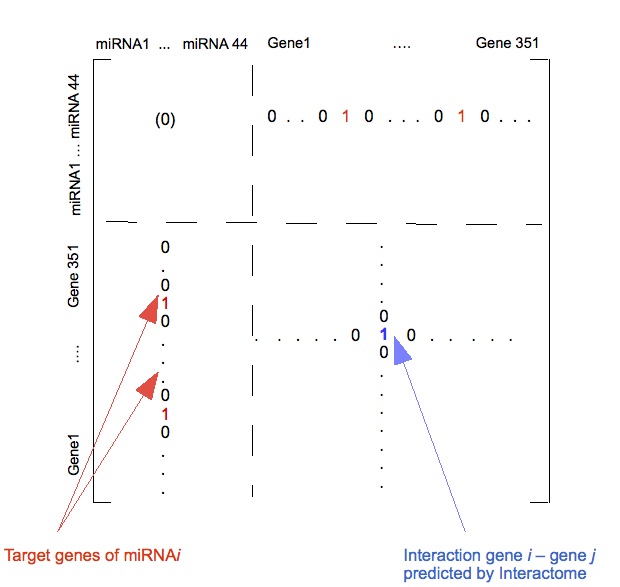


**Figure S4.** **Putative miRNAs encoded by the mitochondrial genome**

A total of 20 putative mature microRNAs were found to be encoded by the mitochondrial genome. Two of these novel miRNAs mapped to the 16S rRNA, 9 mapped to various tRNAs, 2 mapped to genes for complex I subunits, 2 mapped to genes for complex IV subunits, and 2 mapped to genes for complex V subunits. Lastly, 2 potential miRNAs mapped to the D-loop locus.


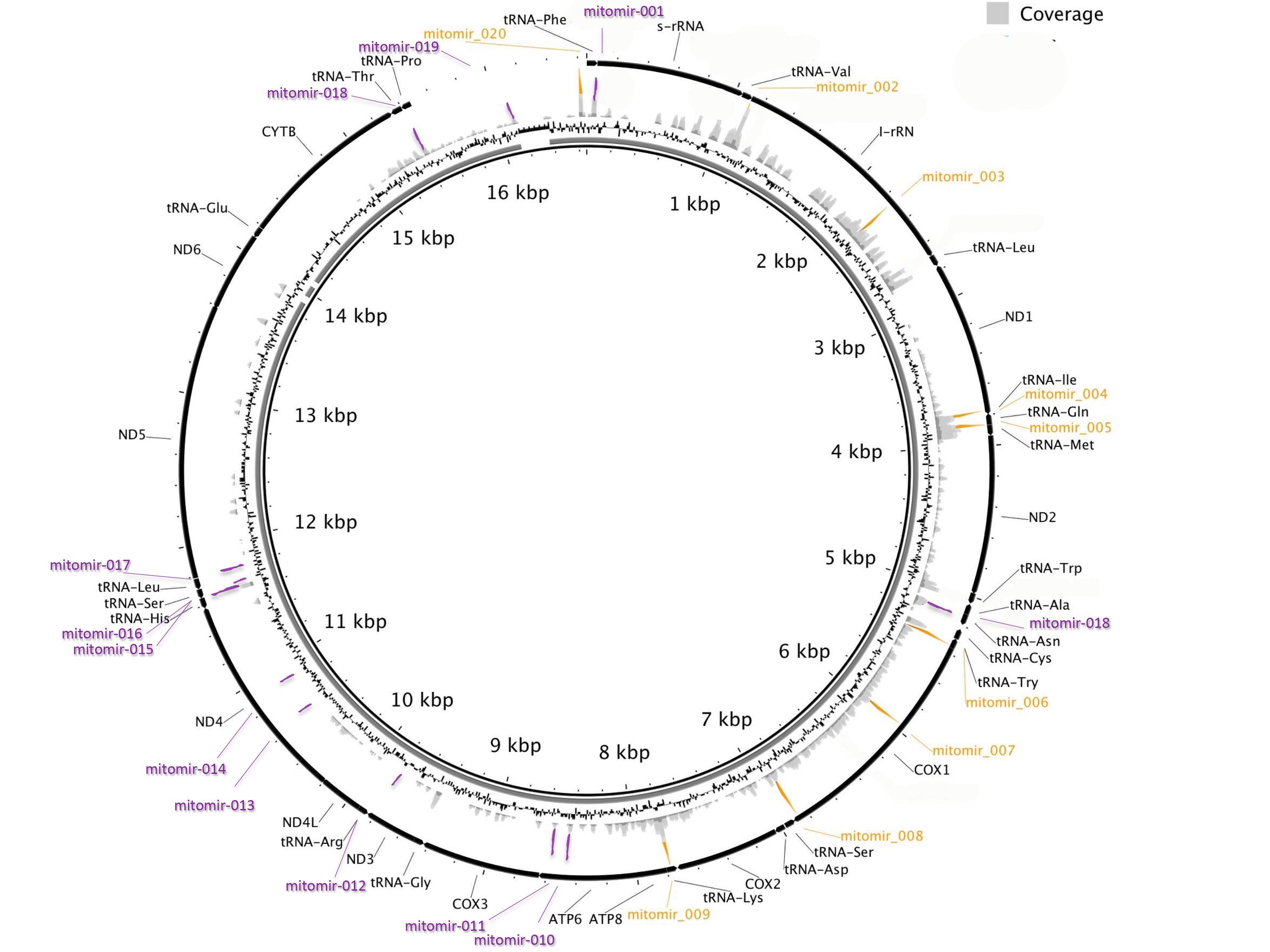


**Figure S5. Relationships between differentially expressed miRNAs, TFs and genes.**

The regulatory network linking differentially expressed TFs, miRNAs and genes was visualized with Cytoscape. The regulatory network showed an overlap between the predicted regulons (*SPI1, FOXO3* and *IRF3*) and the differentially expressed miRNAs. The network is

displayed graphically as being composed of nodes (genes, TF and miRNAs) and edges (biological relationships). The edge colour intensity indicates the expression level of the association: red = over-expression at T1 and green = under-expression at T1. The node shape indicates whether the node is a gene (circles), a TF (triangles) or an miRNA (square).


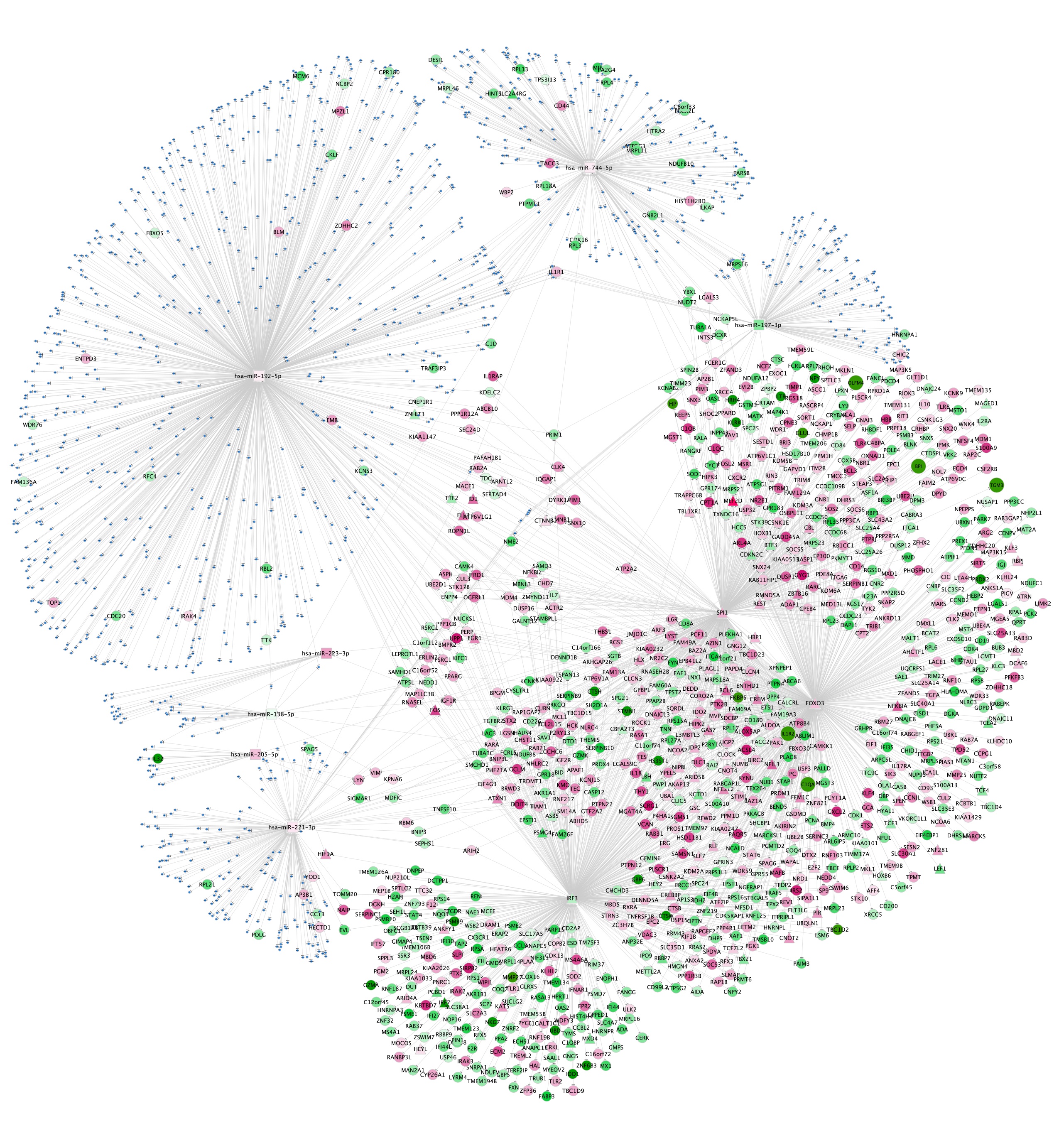


**Figure S6. A functional map of the regulatory network linking TFs, differentially expressed miRNA and differentially expressed genes**

The barplot shows significant biological pathways. The GO biological terms were identified as bars and linked on the basis of their kappa value (>0.4) and FDR (<0.001). Similar GO terms are given in the same colour. Non-grouped terms are shown in grey. Non-grouped terms are shown in grey. The proportion of genes in each cluster associated with the term is described above the bars.

**
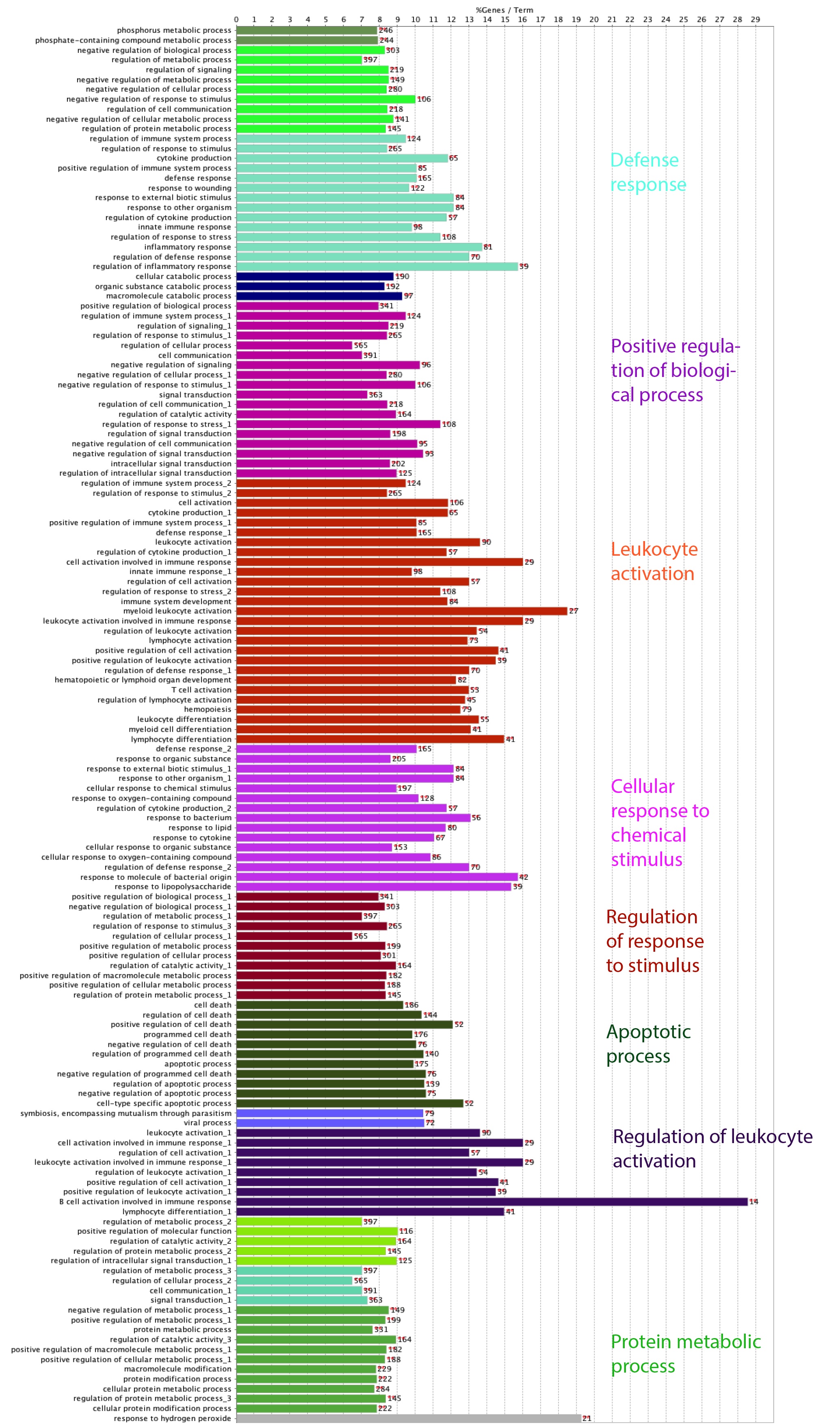
**

**Figure S7**. **Quantitative real-time PCR for selected DEGs** and miRNAs

Genes (A) and miRNAs (B) determined to be differentially expressed when comparing pre- and post-ride samples by microarray analysis were validated by RT-qPCR. For each of the validated genes and miRNAs, boxplots were calculated for 10 animals from the experimental set.


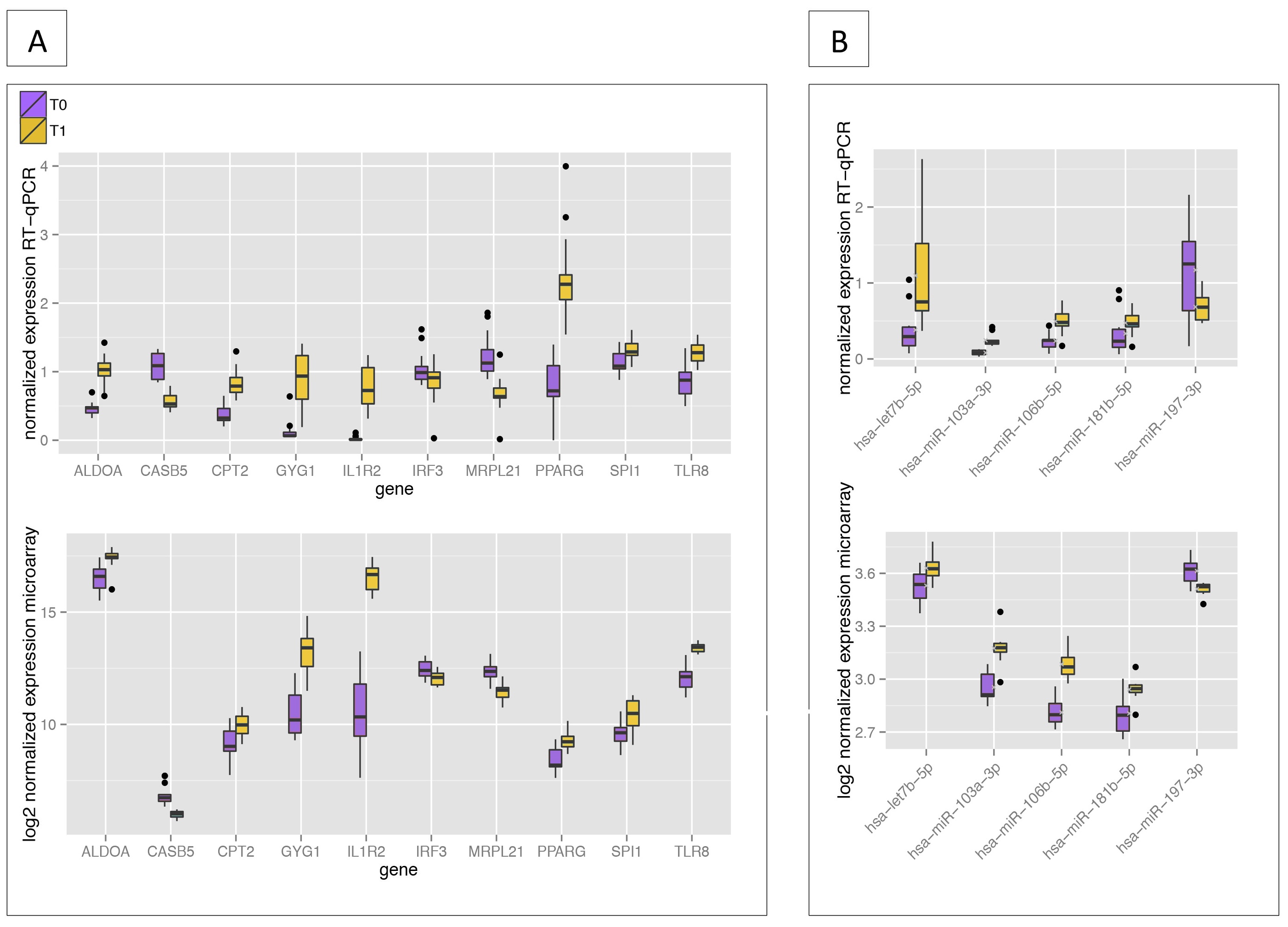


**Figure S8. Generation of the PPI sub-network by mapping 351 depleted target DEGs and their regulatory miRNAs onto the BioGRID parental PPI network.** A) A total of 337 genes presented PPI information and, with their first neighbouring proteins, formed a PPI sub-network containing 6,024 nodes and 21,314 edges. In all cases, green and red nodes respectively represent proteins encoded by under- and over-expressed genes at T1 relative to T0. Blue nodes represent interacting proteins that were not significantly differentially expressed. The node arrangement was applied to the “Spring Embedded” layout in Cytoscape.


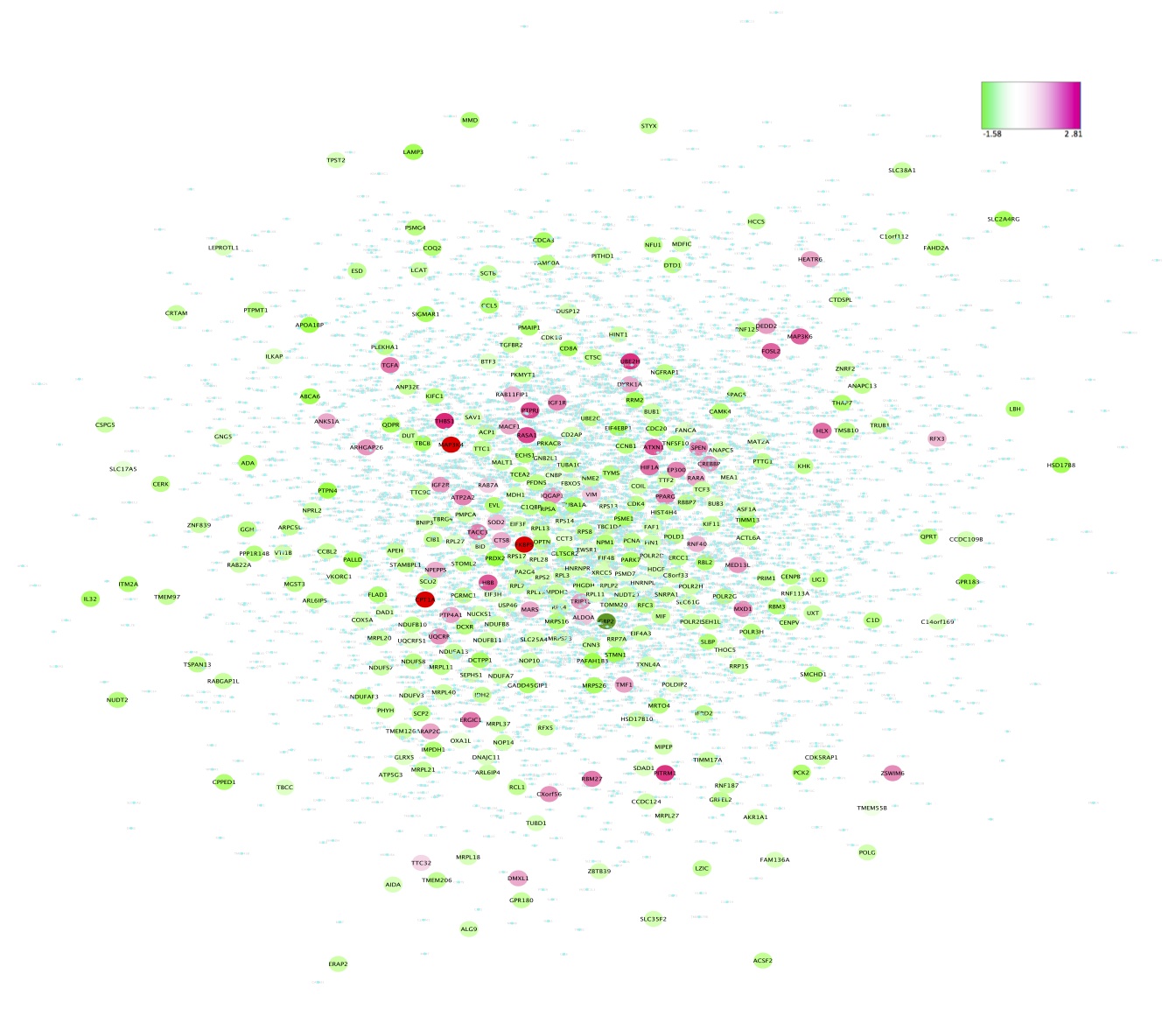


**Figure S9. Interactions between the 351 depleted target DEGs**

The network was created by merging three IPA networks: the first network included genes involved in the cell cycle, small molecule biochemistry and cancer; it presented a score of 29 and had 20 focus genes. The second network was associated with molecular transport, lipoid metabolism and small molecule biochemistry; it presented a score of 23 and had 17 focus genes. The third network featured genes involved in lipid metabolism, small molecule biochemistry, and digestive system development and function; it presented a score of 21 and had 16 focus genes. The network is displayed graphically as nodes (gene/gene products) and edges (biological relationships). The node colour intensity indicates the genes’ expression level: red = over-expression at T1 and green = under-expression at T1, relative to the same animal at T0. The node shape indicate the gene product’s functional class.

**
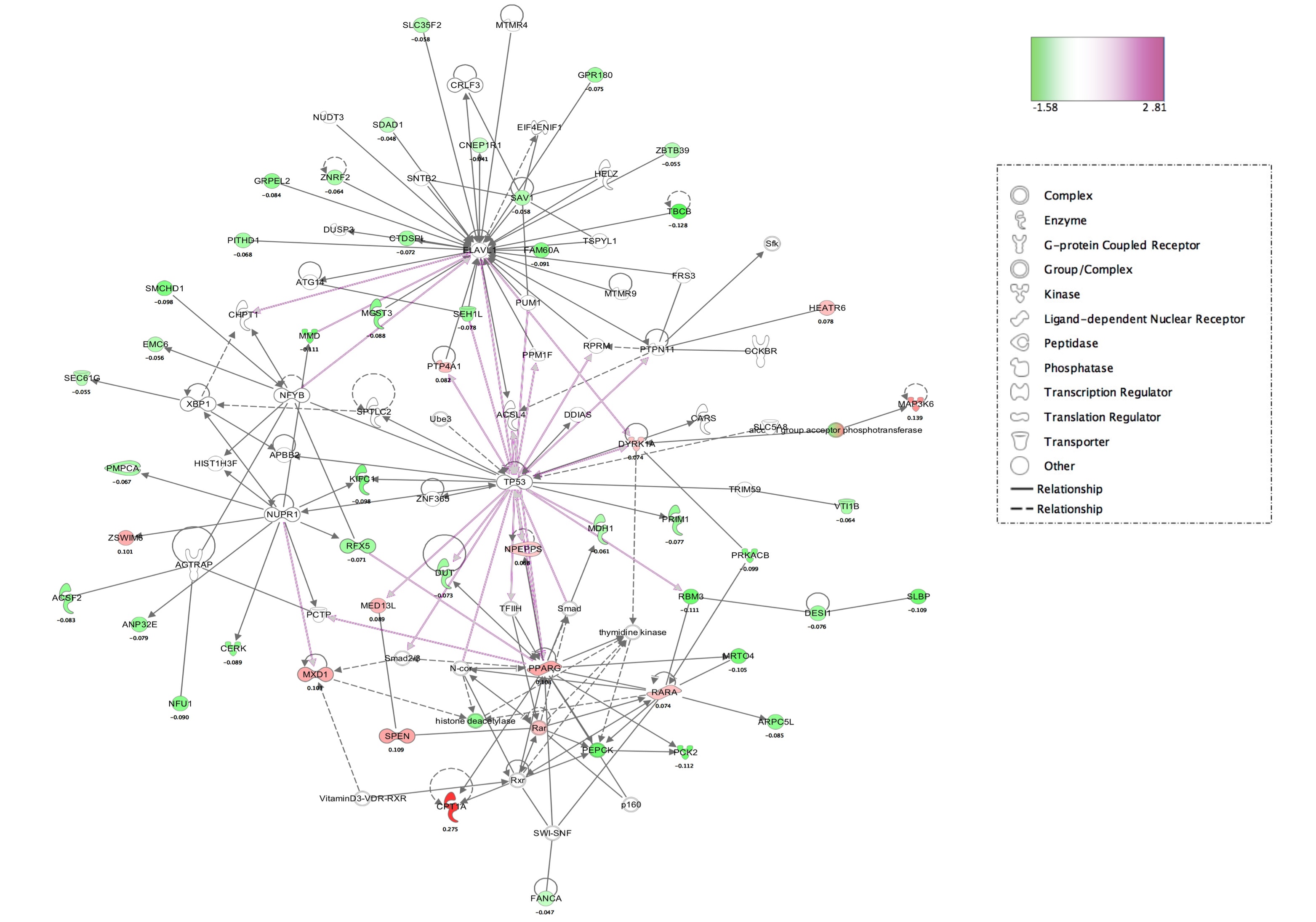
**

**Figure S10.** Correlation matrix for changes in enriched DEmiRNA levels and endurance-exercise-related biochemical blood parameters. In the graph, CK= creatine kinase, ht= haematocrit or the packed cell volume, ASAT= aspartate transaminase, GGT= gamma glutamyltransferase, SAA= serum amyloid A (SAA), PT=total plasma proteins, Bil_Conj=conjugated bilirubin and Bil_Tot=total bilirubin.

**Figure S11.** Correlation matrix for changes in the expression of the differentially expressed miR-133b and miR-133a-3p and endurance-exercise-related blood parameters. Only statistically significant correlations (p < 0.05) are shown in the plot. In the graph, CK= creatine kinase, ht= haematocrit or the packed cell volume, ASAT= aspartate transaminase, GGT= gamma glutamyltransferase, SAA= serum amyloid A (SAA), PT=total plasma proteins, Bil_Conj=conjugated bilirubin and Bil_Tot=total bilirubin.

**Supplementary Tables**

**Table S1. The horses’ morphological and physiological parameters**

**Table S2.** Biochemical parameters obtained from 45 blood samples collected after the 160 km ride (14 horses from the experimental set and 31 horses from the validation set)

**Table S3. DEGS in 14 animals following endurance exercise (a 160 km ride)**

**Table S4. miRNAs present in the blood of horses following endurance exercise**

**Table S5. Differentially expressed miRNAs in 14 animals following endurance exercise (a 160 km ride)**

**Table S6. Biological process terms associated with the differentially expressed genes following endurance exercise**

**Table S7. Experimentally annotated targetome of the differentially expressed miRNAs**

**Table S8. Enriched miRNAs**

**Table S9. Enriched miRNAs with their potential target DEGs**

**Table S10. Expression of 351 target DEGs that were inversely correlated to the enriched DEmiRNAs during exercise**

**Table S11. Estimation of the coefficient of determination (R^2^) and the MSE in GGMs**

**Table S12. Potential novel miRNAs detected by sRNAseq**

**Table S13. Sequences of the gene primers used for the RT-qPCR**

**Table S14. miRNA primers used for the RT-qPCR**

**Table S15. Network topology parameters**
